# Combining skin and olfactory α-synuclein seed amplification assays (SAA)—towards biomarker-driven phenotyping in synucleinopathies

**DOI:** 10.1038/s41531-023-00519-8

**Published:** 2023-05-29

**Authors:** A. Kuzkina, J. Rößle, A. Seger, C. Panzer, A. Kohl, V. Maltese, T. Musacchio, S. J. Blaschke, G. Tamgüney, S. Kaulitz, K. Rak, A. Scherzad, P. H. Zimmermann, J. P. Klussmann, S. Hackenberg, J. Volkmann, C. Sommer, M. Sommerauer, K. Doppler

**Affiliations:** 1 University Hospital Würzburg (UKW), Department of Neurology, Josef-Schneider-Str. 11, 97080 Würzburg, Germany; 2 University Hospital Cologne, Department of Neurology, Faculty of Medicine, University of Cologne, Kerpener Str. 62, 50937 Cologne, Germany; 3 Institut für Physikalische Biologie, Heinrich-Heine-Universität Düsseldorf, 40225 Düsseldorf, Germany; 4 Institute of Biological Information Processing (Structural Biochemistry: IBI-7), Forschungszentrum Jülich, 52428 Jülich, Germany; 5 University Hospital Würzburg (UKW), Department of Oto-Rhino-Laryngology, Plastic, Aesthetic and Reconstructive Head and Neck Surgery, Josef-Schneider-Str. 11, 97080 Würzburg, Germany; 6 University of Cologne, Medical Faculty, Department of Otorhinolaryngology, Head and Neck Surgery, Kerpener Strasse 62, 50931 Cologne, Germany; 7 Center for Molecular Medicine Cologne (CMMC), Faculty of Medicine, University Hospital Cologne, Robert-Koch-Strasse 21, 50931 Cologne, Germany; 8 Institute of Neuroscience and Medicine (INM-3), Forschungszentrum Jülich, Jülich, Germany; 9 Present Address: Ann Romney Center for Neurologic Diseases, Brigham and Women’s Hospital and Harvard Medical School, Boston, MA 02115 USA; 10 Present Address: Division of Movement Disorders, Department of Neurology, Brigham and Women’s Hospital, Boston, MA 02115 USA; 11 Present Address: RWTH Aachen University, Department of Oto-Rhino-Laryngology, Head and Neck Surgery, Aachen, Germany

**Keywords:** Diagnostic markers, Parkinson's disease, Parkinson's disease

## Abstract

Seed amplification assays (SAA) are becoming commonly used in synucleinopathies to detect α-synuclein aggregates. Studies in Parkinson’s disease (PD) and isolated REM-sleep behavior disorder (iRBD) have shown a considerably lower sensitivity in the olfactory epithelium than in CSF or skin. To get an insight into α-synuclein (α-syn) distribution within the nervous system and reasons for low sensitivity, we compared SAA assessment of nasal brushings and skin biopsies in PD (*n* = 27) and iRBD patients (*n* = 18) and unaffected controls (*n* = 30). α-syn misfolding was overall found less commonly in the olfactory epithelium than in the skin, which could be partially explained by the nasal brushing matrix exerting an inhibitory effect on aggregation. Importantly, the α-syn distribution was not uniform: there was a higher deposition of misfolded α-syn across all sampled tissues in the iRBD cohort compared to PD (supporting the notion of RBD as a marker of a more malignant subtype of synucleinopathy) and in a subgroup of PD patients, misfolded α-syn was detectable only in the olfactory epithelium, suggestive of the recently proposed brain-first PD subtype. Assaying α-syn of diverse origins, such as olfactory (part of the central nervous system) and skin (peripheral nervous system), could increase diagnostic accuracy and allow better stratification of patients.

## Introduction

Parkinson’s disease (PD) is associated with the deposition of misfolded α-synuclein (α-syn). Histopathological biomarker studies have revealed widespread accumulation of pathological α-syn in the peripheral nervous system (PNS), which is mainly studied through skin, gastrointestinal, or submandibular gland biopsies^1^. The development of ultrasensitive seed amplification assays (SAA) has opened possibilities for sampling tissues where α-syn pathology was not detectable before^2^. One such example is neuro-olfactory epithelium which can be obtained through a non-invasive nasal brushing procedure^3^, an approach that, in the case of prion diseases, has proven to even be diagnostically superior to the analysis of cerebrospinal fluid^3–5^.

Since hyposmia is a frequent prodromal symptom of PD and Lewy body pathology affects the olfactory system in the early disease stages, assaying olfactory epithelial cells could allow early detection of α-syn pathology in the majority of patients^6,7^. However, the first trials of nasal brushings performed in synucleinopathies were not in agreement with this assumption. In these studies, α-syn seeding activity was detectable in 46–69% of patients with PD^8–10^, 44% of patients with isolated REM-sleep behavior disorder (iRBD)^10^, and 82–90% of patients with multiple system atrophy with predominant parkinsonism (MSA-P)^8,9^.

One reason for the generally low sensitivity could be simply of technical nature, e.g., inefficient sampling of the olfactory epithelium (sampling from a single nostril, incorrect sampling of the olfactory epithelium) and/or properties of the sample matrix, representing a diverse tissue mix some of the components of which could inhibit protein aggregation. Alternatively, the absence of α-syn pathology in the olfactory epithelium could be reflective of differences in α-syn distribution among synucleinopathy patients. Indeed, there appears to be no single pattern of α-syn distribution, as up to half of the neuropathological PD cases show Lewy pathology distribution incompatible with Braak staging^11–13^. Furthermore, growing evidence suggests the existence of two main PD subtypes: a body-first, in whom the Lewy pathology originates in the peripheral autonomic nervous system and spreads to the central nervous system (CNS), and a brain-first, in whom the pathology may start in the amygdala and/or olfactory bulb and descends to the periphery^14,15^. In agreement with this hypothesis, the α-syn deposits in the dermal nerves are detectible in early and prodromal body-first PD; however, they would appear later in the course of brain-first PD. On the other hand, analysis of nasal brushings could allow for earlier detection of α-syn in the latter subgroup.

In this pilot study, we aimed to investigate the distribution of α-syn aggregates in olfactory and dermal nervous tissue at various stages and in different neurodegenerative Parkinsonian diseases. We collected nasal brushings from patients with PD and iRBD, as well as from control subjects. In a subcohort of patients, we additionally sampled skin biopsies to get an insight into the patterns of distribution of α-syn pathology.

## Results

### Study population

This study was performed between 2019 and 2022 and was severely protracted due to the COVID-19 pandemic. A total of 81 participants were included: 27 patients with PD, 18 patients with iRBD, 6 patients with atypical parkinsonism (3 with progressive supranuclear palsy (PSP), 3 with multiple system atrophy (MSA)), and 30 control subjects. Demographic characteristics and main clinical data are summarized in Table 1. The nasal brushing procedure was very well tolerated, participants reported short-lasting discomfort or slight pain during the procedure with an average of 2.6 (±2) on the numerical rating scale (NRS), and there were no adverse events.

**Table 1:** Demographic data and main SAA results.

	PD (*N* = 27)	RBD (*N* = 18)	Atypical parkinsonism (*N* = 6)	Control (*N* = 30)	*P*-value
Sex: female	7 (25.9%)	3 (16.7%)	4 (66.7%)	13 (43.3%)	0.063
Male	20 (74.1%)	15 (83.3%)	2 (33.3%)	17 (56.7%)	
Age, mean (SD)	64.0 (8.78)	65.9 (6.99)	69.4 (4.74)	60.7 (11.1)	0.098
H&Y stage: mean (SD)	2.56 (0.70)	–	3.00 (0.89)	–	0.189
Duration, years: mean (SD)	12.4 (5.62)	8.21 (7.07)	3.33 (2.42)	–	0.003
Positive nasal brushing SAA, *N* (% of sampled)	13 (48.1%)	12 (67%): 8 right-sided (44.4%), 11 (61.1%) left-sided	1/3 MSA, 0/3 PSP	3 (10.0%)	<0.001
Positive skin biopsy SAA, *N*/total: (% of sampled)	15/19 (78.9%)	15/15 (100%)	3/3 MSA, 0/3 PSP	NA	0.113 for PD vs. iRBD
Thigh	12 (70.6%)	14 (93.3%)	3 (60.0%)	NA	0.095 for PD vs. iRBD
Neck (C7)	9 (47.4%)	13 (86.7%)	3 (60.0%)	NA	0.043 for PD vs. iRBD

### Olfactory epithelium seeds α-syn aggregation with high specificity but low sensitivity

**Fig. 1:**
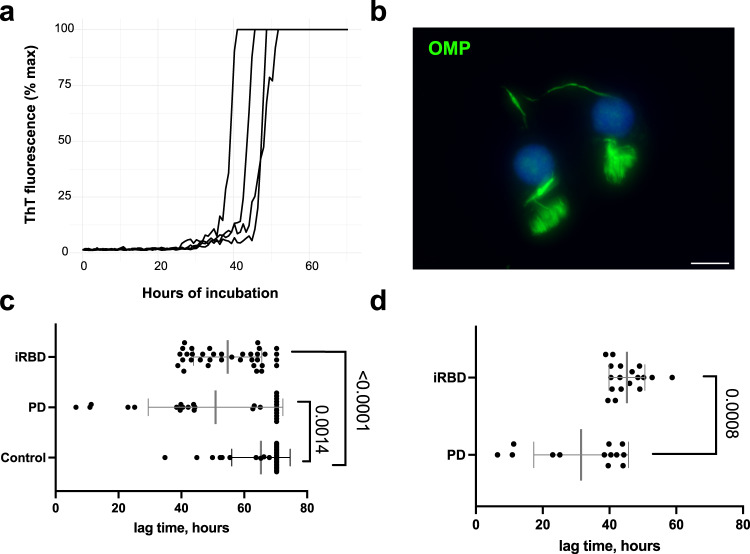
α-syn aggregation in nasal brushing from PD and iRBD patients is readily detectible by SAA, but eludes immunostaining. **a** Representative SAA response seeded by a nasal brushing of a synucleinopathy patient: all four technical replicates are shown, and ThT fluorescence is expressed in percentage of the maximum measurable response. See Supplementary Material for graphs of all samples. **b** Immunohistochemical staining of nasal brushings for olfactory marker protein (OMP, green), scale bar = 10 µm. **c** Lag times of all nasal brushing samples in PD (*n* = 27), iRBD (*n* = 36, samples of both sides of the nasal cavity are plotted), and controls (*n* = 30). Mean (±SD) is plotted for each group. **d** Individual data points and mean values (±SD) of lag times of only positive PD and iRBD samples.

Using nasal brushings as a matrix for SAA resulted in very efficient seeding with typically a simultaneous increase in fluorescence of all four replicates (Fig. 1a). Serial dilutions of nasal brushings revealed different ranges of seeding capacity in the olfactory epithelium of PD patients, as in some samples aggregation persisted up to a 1000-fold dilution, in other it began to wane beyond the initial 1:20 dilution (5 µl of the lysate is directly added to 95 µl buffer) (Supplementary Fig. 1). As the outcomes in the control samples remained virtually the same through the dilution range, we proceeded with the 1:20 dilution, which showed the highest sensitivity.

The average length of the lag phase in positive samples was 43 h. There was a wide range in lag times among samples of different patients (SD = ±14), with three PD samples showing remarkably early aggregation times of less than 15 h (Fig. 1c, d). These samples were re-tested, and the results remained consistent (see Supplementary Material for raw data and graphs of all runs). There was little spontaneous aggregation: negative samples had, on average, 0.5 (±0.8) positive replicates, and in 35 (of total of 39) negative samples, there was not a single positive replicate.

### The nasal brushing matrix has an inhibitory effect on α-syn aggregation, independent of hemoglobin contamination

**Fig. 2:**
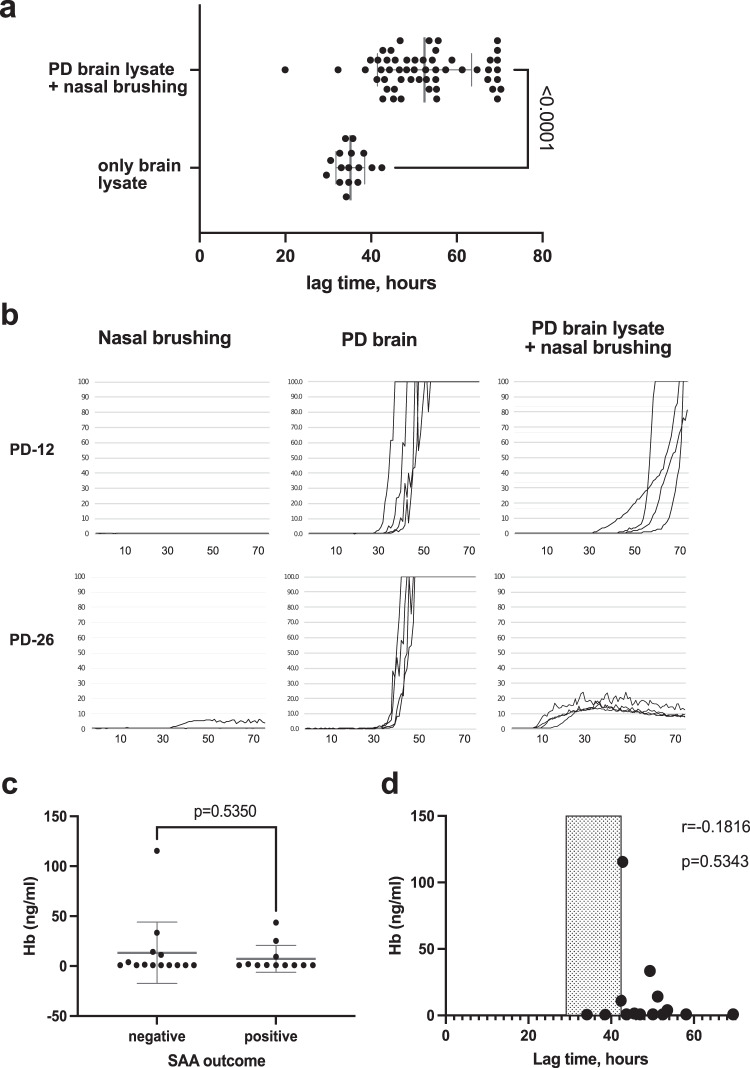
Inhibition of a-syn aggregation by nasal brushing matrix: re-testing of the negative nasal brushing samples (*n* = 50) after spiking with PD brain lysate. **a** The lag times in the SAA seeded by both nasal brushings and brain lysate (*n* = 50) compared to the same brain lysate tested alone (*n* = 15 separate assays), each dot represents the mean of 4 replicates. Mean values (±SD) are plotted in gray for each group. **b** Examples of lag-phase prolongation and change in ThT curve morphology in the PD brain lysate aggregation caused by the addition of some of the nasal brushings. See Supplementary Fig. 2 for further examples. **c** Mean (±SD) of Hb concentration in the PD nasal brushings at the assay-relevant dilution (1:20) grouped based on the SAA outcome. **d** Correlation of Hb concentration with the lag time of samples spiked with PD lysate. The typical range of the lag times in the brain lysate alone is shaded.

Given the surprisingly low nasal brushing sensitivity in PD and iRBD, we retested negative nasal brushing samples after spiking them with the positive control (midbrain lysate, Braak stage 3) to check for the potential inhibitory effect of the sample matrix on α-syn aggregation. All negative control (*n* = 27) and PD samples (*n* = 14) and the majority of negative iRBD samples (*n* = 9) were re-tested with and without the addition of a positive control seed, in total *n* = 50 (91% of all negative samples). The results (without spiking with PD brain lysate) remained consistently negative in all these samples. The addition of the nasal brushings to the PD brain sample significantly prolonged the lag phase (Fig. 2a); in several cases (*n* = 12, 24%), the α-syn aggregation seeded by brain lysate was completely inhibited (Supplementary Fig. 2B), and in five (10%) cases it markedly changed the ThT curve morphology (Fig. 2b and Supplementary Fig. 2C).

We assessed all PD samples, *n* = 27, for the presence of hemoglobin (Hb) as an indicator of blood contamination that may affect α-syn aggregation^16,17^. Hb ELISA revealed overall low levels of Hb in the samples, averaging at 10.16 ± 23.7 ng/ml (the samples below the detection limit of 0.8445 ng/ml, *n* = 11, were considered equal to 0.8445 for this calculation). There was no relevant difference between Hb levels in the SAA-positive and -negative samples (Fig. 2c) and no correlation of the length of the lag phase in the brain lysate spiked by the according nasal brushing sample with its Hb content (Fig. 2d).

### Nasal brushing SAA yields slightly different results in PD and iRBD groups

In the PD cohort, α-syn seeding was detectable in the one-sided nasal brushings of about half of the patients (*n* = 13, 48%). In the sampling of the right side of the nasal cavity, 44% of iRBD patients were positive, 61% in the sampling of the left side, and 67% of iRBD patients (12 out of 18 patients) had positive SAA reaction in either of the samples, while 7 (39%) were positive in both. The lag times in the positive samples were significantly shorter in the PD cohort (31.5 (±14.2) hours compared to 45.2 (±5.4) h in iRBD, *p* = 0.0008). The maximum fluorescence values in the PD cohort (72.8% RFU (±32.5)) were not significantly different from iRBD (90.5% RFU (±11.6), *p* = 0.085).

In the small cohort of patients with atypical parkinsonism, only one MSA (parkinsonian type) patient was positive. The maximum fluorescence (13%RFU) was considerably lower compared to PD and iRBD patients.

Three (10%) control samples were positive, with a lag time of 46 h (±14) and maximum fluorescence of 89% RFU (±2.4). These control samples were tested again due to an unexpected result and remained positive.

### Comparison to skin biopsy

In the subgroup of PD (*n* = 19) and iRBD patients (*n* = 15) that received both skin biopsies and nasal brushings, α-syn seeding was more commonly seen in skin biopsies (see Supplementary Table 2). Within this subcohort, 15 (78.9%) PD patients had at least one positive skin biopsy, while only 7 (37%) showed α-syn seeding in the nasal brushing. However, three PD patients with absent seeding in the skin biopsies demonstrated positive α-syn SAA in the nasal brushings. If combined, nasal brushing and skin biopsy identified α-syn seeding in 18/19 (95%) PD patients, with the only negative patient having a 1-year disease duration. In the iRBD cohort, α-syn seeding was detectable in the skin of all 15 biopsied patients and in 10 (67%) of the corresponding nasal brushings. All three MSA patients were positive in the skin biopsy and one in the nasal brushing; none of the three PSP patients were positive in either nasal brushing or skin biopsy.

### Pathological α-synuclein is not detectable using immunostaining

Double immunostaining for olfactory marker protein (OMP) and anti-S129-phosphorylated α-syn (anti-p-α-syn) was performed for all nasal brushings collected in Würzburg. No p-α-syn positive depositions in olfactory neurons were found. OMP-positive olfactory neurons with typical morphology (Fig. 1b) could be found in all cytospin preparations of nasal brushings, although the yield varied, with a mean of 1.8 (±0.6) on the scale from 0 to 3 (see “Methods”). The OMP-staining results did not differ between SAA-positive and negative samples (*p* = 0.75).

### Correlations with clinical markers of disease

There was no correlation of the binary nasal brushing SAA outcome with any of the examined clinical markers in either PD or iRBD subgroups (see Table 2). However, a higher number of totally assessed samples positive for α-syn (sum of positive samples varying from 0 to 3, including nasal brushing (0–1), neck biopsy (0–1), and thigh biopsy (0–1)) in PD and iRBD was associated with a worse olfactory function (2.29 (±0.76) vs. 2.06 (±0.94) vs. 0.75 (±0.5) in anosmic, hyposmic and normosmic patients respectively, *p* = 0.02). In PD higher number of positive samples was also associated with the presence of probable RBD (1.80 (±0.63) vs. 1.12 (±0.64), *p* = 0.041) and with complaints about constipation (2.17 (±0.41) vs. 1.14 (±0.53), *p* < 0.001) or any gastrointestinal problems (questions 4–7 of the NMSQ) (2.00 (±0.50) vs. 1.00 (±0.45), *p* < 0.001). The iRBD group had a significantly higher burden of α-syn depositions with, on average, 2.47 (±0.83) positive samples compared to 1.45 (±0.69) in PD, *p* = 0.001. In the iRBD subgroup, there was no lateralization of the side of positive nasal brushing with the DaTscan data or MDS-UPDRS motor assessment.

**Table 2:** Characteristics of patients with positive and negative nasal SAA.

	Parkinson’s disease			iRBD		
	negative nasal SAA, *N* = 14	positive nasal SAA, *N* = 13	*p* value	negative nasal SAA, *N* = 6	positive nasal SAA, *N* = 12	*p* Value
*Sex*			0.678			0.515
Female	3 (21.4%)	4 (30.8%)		0 (0.00%)	3 (25.0%)	
Male	11 (78.6%)	9 (69.2%)		6 (100%)	9 (75.0%)	
Age, years (SD)	62.2 (9.95)	65.9 (7.22)	0.283	65.5 (9.80)	66.1 (5.62)	0.892
Disease duration, years (SD)	12.6 (5.49)	12.2 (5.98)	0.827	10.3 (9.97)	7.15 (5.32)	0.490
H&Y stage, mean (SD)	2.50 (0.52)	2.62 (0.87)	0.683	NA		
DBS surgery, *n* (%)	10 (71.4%)	7 (53.8%)	0.440	NA		
Skin SAA positive, *n* (% of biopsied)	11/12 (91.7%)	4/7 (57.1%)	0.117	18/18 (100%)		
*Subtype, n (%)*			0.282	NA		
Akinetic-rigid	11 (78.6%)	7 (53.8%)				
Mixed	3 (21.4%)	4 (30.8%)				
Tremor-dominant	0 (0.00%)	2 (15.4%)				
Sniffin Sticks: TDI for PD, screening for iRBD	19.4 (7.86)	18.9 (5.09)	0.870	7.33 (4.13)	5.58 (3.00)	0.384
Positive RBD screening, *n* (%)	7 (50.0%)	5 (38.5%)	0.830	NA		
NMSQ sum (SD)	8.29 (2.33)	11.4 (5.80)	0.136	5.83 (5.78)	6.58 (2.91)	0.774
MDS-UPDRS part 1, sum (SD)	5.29 (3.75)	8.30 (4.99)	0.126	4.20 (3.19)	5.60 (3.69)	0.467
MDS-UPDRS part 2, sum (SD)	14.1 (5.38)	12.1 (5.63)	0.400	1.00 (1.73)	1.40 (1.51)	0.673
MDS-UPDRS part 3, sum (SD)	NA^a^			5.17 (2.48)	3.08 (2.19)	0.115
MDS-UPDRS part 4, sum (SD)	3.93 (4.29)	4.62 (5.28)	0.715	NA		
MoCA, sum (SD)	27.3 (1.94)	27.5 (2.02)	0.748	28.0 (2.00)	27.7 (1.78)	0.737
RBDSQ sum (SD)	NA			10.2 (2.40)	10.2 (2.17)	1
Orthostatic hypotension, tested in the clinic	NA			2 (33.3%)	4 (33.3%)	1
Pathological DaTscan	NA			1/2 (50.0%)	6/8 (75.0%)	1

^a^A standardized MDS-UPDRS3 assessment in an off-state was not feasible in the PD cohort.

Of particular interest is the small subgroup of PD patients (*n* = 3) in whom α-syn seeding could be detected in nasal brushing but not in skin biopsies. The burden of non-motor symptoms was significantly lower in these patients than in the rest of the PD patients (NMSQ sum of 5.33 (±0.58) vs. 9.93 (±4.53), *p* = 0.002). None of these patients reported RBD symptoms or any gastrointestinal problems (see Supplementary Table 1 for a more detailed overview). These patients did not significantly differ in disease duration or age from the rest. All three patients were genetically tested for 68 PD-associated genes with a negative result (in one, a PINK1 variant of unknown significance was detected)^18^.

All three control subjects positive for nasal SAA were anosmic in formal testing (in one case, due to chronic rhinosinusitis, in two other cases, no reason for anosmia was known, and the probands were not aware of having anosmia prior to formal testing), and one control subject additionally screened positive for iRBD. None of the control subjects could be diagnosed with prodromal PD based on the screening results^19^.

## Discussion

In this pilot cross-sectional study, we investigated the presence of misfolded α-syn in nasal brushings of patients with PD and iRBD while directly comparing the findings to the skin biopsies. Using SAA, we detected α-syn seeding in the nasal brushings of 48% of PD, 67% of iRBD patients, and 10% of control subjects. The sensitivity of the nasal brushing was inferior compared to the skin SAA, where all the biopsied iRBD patients and 79% of PD patients were positive.

While the low sensitivity of the nasal brushings is in line with previously reported studies^9,10^, we uncovered several new important aspects. This is the first study to simultaneously assess skin biopsy and nasal brushings. These tissues relate to distinct origins of misfolded α-syn: skin-derived α-syn predominantly originates from the autonomic PNS^20,21^, while nasal brushing gives access to the olfactory neuroepithelium, or the cranial nerve I, whose axons terminate in the olfactory bulb (and the whole olfactory system is considered part of CNS). As olfactory and autonomic nervous systems are supposed to be the two earliest sites in the spread of Lewy pathology^22^, simultaneously studying the misfolded α-syn from both tissues uniquely positions us to glean an insight into the in vivo α-syn distribution.

According to the long-standing dual-hit hypothesis, Lewy pathology invariably spreads to the CNS simultaneously through the nasal and enteric routes^22,23^. Later studies demonstrated that up to half of post-mortem cases do not fit into this model^11–13^, which possibly gets reflected in the remarkable diversity of clinical manifestations in PD^24^. Neuropathological evidence has been recently systematically revised in the α-synuclein Origin site and Connectome (SOC) model^25^. According to the SOC model and substantiated with in vivo imaging data, PD can be subtyped into brain-first and body-first^14^. In the brain-first subtype, pathology initiates in the amygdala and/or olfactory system with later involvement of substantia nigra and, finally, the lower brainstem and PNS. In the body-first subtype, pathology starts in the peripheral autonomic nervous system and ascends to the brainstem. Accordingly, body-first patients present with prominent autonomic symptoms and RBD in the prodromal stage, while brain-first patients present with motor symptoms and develop autonomic symptoms much later^26^. In this context, we can presume that our iRBD cohort is represented by body-first patients, while the PD group includes both subtypes.

We attempted to look at our data from the perspective of known neuropathological evidence, in particular correlating it to the recently proposed brain-first and body-first neuropathological subtypes. We recognize that any conclusions from our data are speculative and see the following main limitations: the absence of α-syn seeding from a nasal brushing does not exclude α-syn pathology in the neuroepithelium, as false-negatives are possible (addressed in detail below); clinical subtyping of PD into the brain- and body-first cannot be definitively achieved in our study, as on the one hand, we do not have sufficient imaging data (i.e., cardiac innervation with MIBG-SPECT) and on the other hand we have also included patients in advanced disease stages, at which point the groups would not be distinguishable as their neuropathological trajectories converge. Nevertheless, some differences detected in α-syn distribution between the iRBD and PD groups and within the PD group deserve recognition.

Firstly, noteworthy differences in the pattern of α-syn aggregate distribution were seen between the PD and iRBD groups. The iRBD patients were almost uniformly positive for both skin biopsy and nasal brushings, with only a few patients who had α-syn aggregates detectible in the skin but not in the olfactory epithelium. Although designated as “body-first”, iRBD patients already have CNS (brainstem) pathology as they are diagnosed, since the phenotype of RBD becomes apparent when the coeruleus/subcoeruleus complex is affected^27^.

As it is known that 20–40% of locus coeruleus neurons project to the olfactory bulb (based on rodent studies)^28,29^, the presence of misfolded α-syn in the olfactory epithelium is thus in line with a potential retrograde spread from the locus coeruleus to the olfactory system in iRBD patients. Overall, there were more total positive samples of skin and nasal brushings in the iRBD group compared to PD. The proportion of samples positive for α-syn SAA was also higher in PD patients who reported RBD symptoms in agreement with prior studies^14,30,31^. The correlation of the extent of α-syn spreading throughout the body with the severity of the NMS fits into the proposed diffuse malignant and body-first phenotypes^26^.

**Fig. 3:**
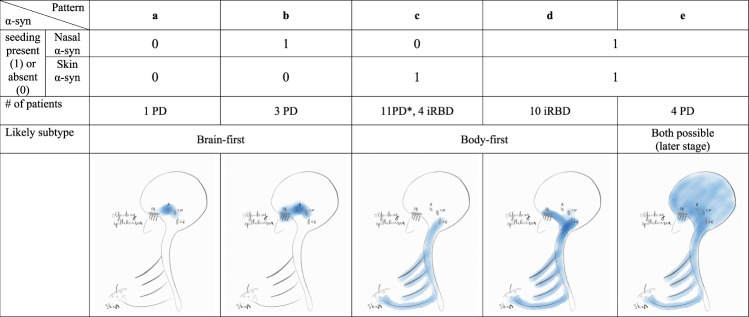
A proposed neuropathological explanation for the found patterns of α-syn distribution. **a** In the brain-first subtype, α-syn pathology could initiate in the amygdala (**a**) and spread retrogradely to the olfactory bulb (OB), explaining a negative finding in both skin biopsy and nasal brushing in the earliest stage of brain-first PD (in our study only one PD patient was allocated to this group; notably the nasal brushing could be a false negative due to pronounced inhibitory effect in this sample). Alternatively, the initial site could be olfactory epithelium. In this case, the absence of α-syn seeding in the nasal brushing would mean a false-negative result. **b** Presence of α-syn seeding in the nasal brushing but not in the skin corresponds to a brain-first PD before pathology spreads caudally. **c**, **d** In the body-first PD, which would include all iRBD patients and a proportion of PD patients, α-syn seeding can be detected in the skin with or without the involvement of olfactory epithelium. The number of PD patients in group C was probably overestimated, as likely more PD patients would have demonstrated α-syn seeding in the nasal brushings if sampled bilaterally. **e** If the pathology is present in both skin and olfactory epithelium in PD, the initial site of α-syn pathology can no longer be determined. SN substantia nigra, LC locus coeruleus.

While nasal brushings had modest sensitivity for α-syn aggregation overall, in a small group of PD patients, α-syn seeding was readily detectable, while concomitant α-syn pathology in the skin (which was otherwise detectable in >90% of PD patients) was absent. If confirmed in a larger cohort in future studies, this finding could have important implications. Firstly, it allows a biomarker-based confirmation of synucleinopathy that would have otherwise been “missed” by the skin biopsy alone. Secondly, the absence of α-syn pathology in the peripheral autonomic system (in this case, skin), together with a relatively benign disease course in this subgroup (few NMS) could imply that we might have identified brain-first PD patients before the pathology spreads to the PNS. Given that α-syn was also detectable in the nasal brushings of the iRBD group, postulated to represent the prodromal phase of the body-first subtype of PD^26^, α-syn pathology in the olfactory epithelium appears to be an early finding in both disease subtypes. Taken together, although nasal SAA does not allow subtyping the patients on its own, it might become feasible in combination with a skin biopsy if confirmed in a larger cohort. We further explain our findings from the perspective of the neuropathological model of the brain- or body-first PD in Fig. 3.

The presence of α-syn pathology in the olfactory epithelium did not correlate with any clinical parameters, including hyposmia. Importantly, the possibility of false-negative samples could have interfered with the correlative analysis. A robust correlation with olfactory dysfunction was previously reported in iRBD^10^ (but not PD) when a larger cohort was studied (*n* = 63). Our study included a significantly smaller iRBD cohort (*n* = 18), the majority of whom (*n* = 12) were positive in nasal SAA, possibly preventing us from seeing any correlation between the clinical data with a binary SAA outcome. More recently^32^, similarly to our observations, it was demonstrated that a significant number of PD patients with preserved olfaction tested positively for nasal SAA. This could indicate that hyposmia has a different pathoanatomic basis in the iRBD and PD groups, i.e., neuro-olfactory epithelium in iRBD and more central pathology in (a subgroup of) PD. Indeed, there is some neuropathological evidence that indicates that total brain α-syn pathology and/or cortical involvement correlates with reduced olfactory function in PD, while Lewy pathology in the olfactory bulb does not^33,34^. A much more complex underlying pathobiology than α-syn misfolding in olfactory epithelium per se is also supported by the commonly known relative sparing of olfaction in MSA, while α-syn seeding is found in the majority of these patients, in up to 90% according to a recent study^8^, and by limited correlation of Lewy pathology to neuronal loss in PD post mortem brains^35–37^.

Remarkably, we did not find Lewy pathology in the olfactory neurons stained for p-α-syn in any of the patients. This can either be explained by a very uneven spread of α-syn pathology among olfactory neurons or seeding by minute quantities of fibrillary α-syn that escape detection by immunocytochemical analysis. Indeed, the life span of mammalian olfactory neurons is reported to be between 15 and 90 days^38^, probably not allowing the time for a build-up of larger protein aggregates that could be visible on immunostaining in the form of Lewy bodies/neurites similar to those seen in the brain and other tissues. While using immunohistochemistry for quality control, we did not find a smaller number of olfactory neurons in patients with negative SAA results. It was recently shown that the sensitivity of α-syn detection differed from 45% in the middle turbinate (concha) to 84% when sampled more superiorly—from the agger nasi, correlating with the abundance of olfactory neurons in the two regions^32^. Indeed, according to a pathological study, olfactory mucosa was present in less than 20% of biopsies from middle turbinate^39^, so this anatomic site should not be routinely used. In both centers participating in our study, however, extra care was taken to sample mucosa from agger nasi and superior concha, so the lower sensitivity cannot be explained by the inappropriate location of the brushing. Such factors as challenging individual anatomy of the nasal cavity or a type of cytological brush could still play a role. Given the untrivial yet non-invasive procedure, in future studies, the nasal cavity should be sampled bilaterally, and more quantitative methods for quality control, e.g., flow cytometry or RT-PCR for OMP, should be implemented.

Of high and until now not enough stressed importance are the effects of the matrix on the SAA results. The addition of negative nasal brushings significantly increased the lag times of α-syn aggregation in the PD brain sample and, in extreme cases, to the extent that the positive control samples were deemed false-negative. We assessed for blood contamination as the most well-known interference source but could not find relevant Hb contamination using ELISA. For CSF, RBC levels of 1000–1250/µl were suggested as relevantly interfering with protein aggregation^16,17^, though according to the only CSF α-syn study, no amount of RBC contamination found in CSF samples could prolong the lag times enough to change the assay outcomes. As our samples have already been lysed, we reverted to hemoglobin (Hb) concentration. At the upper level of the normal blood mean corpuscular hemoglobin (MCH) of 31 pg/RBC, the RBC count of 1000/µl corresponds to 31 ng/µl of Hb in the sample, or for the reported^16,17^ CSF dilutions (1:6–1:10) in the range of 3000–5000 ng/ml Hb in the final SAA buffer. The maximum level we could detect in our samples was over 20-fold lower (115 ng/ml, the mean was 10 ± 24 ng/ml). Finally, there was no significant difference in the Hb levels between SAA-positive and negative samples, which makes blood contamination an unlikely source of interference. It should be noted that Hb ELISA has not been validated for this tissue, and red blood cell (RBC) counts should be attempted in fresh samples to exclude this contamination source conclusively. However, in contrast to lumbar puncture, nasal brushings are inherently non-invasive procedures, and we have not encountered any procedure-associated bleeding in our subjects. An alternative explanation could be the presence of mucins in the epithelial samples^40,41^, which were previously reported to inhibit prion aggregation^42^. Intriguingly in some cases, the addition of nasal matrix flattened the ThT-tracked aggregation curves, resulting in curve configuration reminiscent of the patterns described for MSA^43^. This effect appears to be relevant primarily for this matrix, as it has not been reported for other matrices (nor previously seen by us), and stresses the limited reliability of the indirect aggregation read-out through ThT fluorescence and the importance of further optimization of the assay (e.g., isolation of α-syn seeds from the matrix^44^).

Our study included a very small group of six patients with atypical parkinsonism (three with MSA and three with PSP). Among them, only one MSA-P patient showed α-syn seeding in nasal brushing SAA, showing a distinctively lower fluorescence signal than seen in PD and iRBD, as was previously shown for CSF^43^. This means that pathological α-syn can be found in the olfactory epithelium in MSA and, as suggested by previous studies^8^, is likely prevalent. Though due to a very small sample size, we cannot contribute any data on the sensitivity of this approach. Nonetheless, nasal brushings could represent an attractive non-invasive alternative SAA matrix for differential diagnosis of PD and MSA^43,45^.

We detected α-syn aggregation in 10% of control subjects, who reported some symptoms that could be attributable to prodromal PD. However, none of them reached a diagnostic certainty level needed to diagnose prodromal PD according to current research criteria^19^. These results are similar to those previously reported^10,32^ and are in line with the frequency of incidental Lewy body disease (10–12%) found post-mortem in the population of >60-year-olds^46^. Larger prospective studies of control subjects could be of value, particularly given the non-invasiveness of nasal brushings.

In summary, pathological α-syn is found in the olfactory epithelium in synucleinopathies with potentially differing neuropathologic trajectories, such as iRBD (hypothesized premotor body-first PD), and across a heterogeneous PD population. Given that neuropathological studies predict olfactory epithelium to be one of the initial sites of α-syn pathology^22^; and for a substantial proportion of patients, likely the only site of pathology in the early stage^47^, it holds the promise of early detection of α-syn misfolding and more effort should be invested to overcome the current limitations. A combination of biomarkers, such as skin biopsies with nasal brushings, would not only result in more efficient α-syn detection but could lead to more precise in vivo neuropathology-based subtyping of patients^48^. The current study’s conclusions are limited by modest sample size and matrix interference with α-syn aggregation, which should be taken into account when planning future research. Further studies allowing for larger recruitment, preferentially of early PD patients with deep phenotyping including RBD-assessment and cardiac MIBG imaging and parallel sampling of multiple tissues (CSF, skin, nasal brushings) will shed more light on the usefulness of combined sampling for clinical practice and subtyping of PD.

## Methods

### Recruitment and clinical assessment

Nasal brushings were acquired from patients with PD, *N* = 27, atypical parkinsonism (multiple system atrophy, *N* = 3, and progressive supranuclear palsy, *N* = 3) and control subjects, *N* = 30, who were recruited at the Department of Neurology at the University Hospital Würzburg. iRBD patients (*N* = 18) were recruited at the University Hospital Cologne. The study was approved by the respective ethics committees, and written informed consent was obtained from all participants. Only PD patients reaching an established degree of clinical diagnostic certainty per MDS criteria were included^49^. The iRBD cohort consisted of patients with a polysomnography-confirmed diagnosis of iRBD^50^. Control subjects did not have any history of parkinsonism or dementia and showed no signs of parkinsonism on neurological examination but could have other unrelated neurological diseases. PD and iRBD patients were assessed using the non-motor symptom questionnaire (NMSQ)^51^ and Unified PD Rating Scale^49^. PD and control patients were screened for probable RBD using a single-question screen^52^. Control and iRBD cohorts were assessed for prodromal PD signs, and prodromal disease risks were calculated^19^. Participants received olfactory testing using Sniffin’ Sticks (an extended test in Würzburg and a screening version in Cologne). FP-CIT-SPECT was performed in 10 patients with iRBD as part of a routine diagnostic workup. The majority (*n* = 40, 78%) of parkinsonism and iRBD patients also received a skin biopsy.

### Olfactory mucosa and skin biopsy sampling procedures

Nasal brushings were performed by experienced otolaryngologists using a rigid endoscope. In both centers, preliminary testing with participating otolaryngologists was performed to ensure correct mucosa sampling (verified by the presence of olfactory epithelium in the brushings). Topical anesthesia consisting of lidocaine plus xylometazoline was applied in the form of intranasal spray or swab prior to endoscopy. A nasopharyngeal flocked swab (Thermo Scientific) was brushed at the olfactory region in the superior nasal concha and submerged into a 5 ml normal saline solution. In Würzburg (PD and control cohorts), only unilateral sampling of the most easily accessible side of the nasal cavity was performed; in Cologne (iRBD cohort), the sampling was performed from both sides. Immediately after the procedure, the participants in Würzburg were asked to evaluate the degree of pain or discomfort during nasal brushing on a scale from 0 (no discomfort) to 10 (maximum pain).

The presence of neuro-olfactory epithelium in the brushings in Würzburg was confirmed by staining for OMP (Thermo Fisher, OSR00037W) of cytospin preparations of an aliquot of the cell suspension (100 µl pro slide). The rest of the cell suspension was aliquoted (1 ml per vial) and pelleted at 2000*g* for 20 min at 4 °C.

The skin biopsy was performed with a 5-mm biopsy punch on one side at the proximal thigh and paravertebrally at C7, as previously described^20^. The biopsies were divided into two equal parts, and only one-half was processed for this study. In 12 cases, the other half was used for an earlier skin SAA study^53^; in other cases, it was used for immunofluorescence as part of a currently ongoing study. Skin material for SAA was flash-frozen in liquid nitrogen, blindly coded, and stored at −80 °C.

### Lysate preparation

Nasal brushing pellets were homogenized using a sonicator (Sonopuls HD 4100, Bandelin) in 200 µl phosphate-buffered saline (PBS) supplemented with protease inhibitors and 0.1% sodium dodecyl sulfate. Sonification was performed on ice in 5-min cycles of alternating 20-s sonication and 10 s pause. The sonication was repeated until no visible debris remained or for a maximum of three 5-min-cycles (energy doses 30–60 kJ). After a 5 min centrifugation at 2000g at 4 °C, the supernatant was filtered through a 0.45 µm polyvinylidene fluoride membrane (Whatman, Cytiva) to clear any remaining debris, aliquoted and stored at −80 °C. 5% w/v skin biopsy lysates were prepared as previously described^53^.

### Immunocytochemistry of nasal brushings

Cytospin preparations (100 µl initial cell suspension per slide) were fixed in 4% paraformaldehyde (PFA) or acetone and stored in PBS at 4 °C for a maximum of one month before staining. Immunocytochemical staining with antibodies against anti-phosphorylated α-syn and OMP was performed (two slides were stained per patient, one acetone- and one PFA-fixed). OMP stain was assessed semiquantitatively by two independent raters at 40× magnification. The presence of OMP-positive olfactory cells was rated with a score from 0 to 3, where 0 meant no OMP positive cells, 1—singular OMP+ cells, but not in every field of view, 2—some OMP positive cells were present in every field of view, and 3—more than five OMP+ cells visible in every field of view.

### SAA substrate

A bacterial plasmid carrying the human α-syn Y136TAT gene (resulting in a wild-type protein sequence) and 6xhistidine-tag at C-terminal (kindly provided by Professor Roucou, University of Sherbrooke, Canada) was used^54–56^. The protein was overexpressed in BL21(DE3) *Escherichia coli* cells. Bacteria were grown at 37 °C to OD_600nm_ of 0.7 in LB media before the expression was induced for 2 h upon the addition of 0.44 mM isopropyl β-d-1-thiogalactopyranoside. The bacterial pellets were resuspended in lysis buffer containing 300 mM NaCl, 50 mM sodium dihydrogenphosphate (pH 7.4), 1 mM phenylmethylsulfonyl fluoride, 0.1 mM tris-(2-carboxyethyl) phosphine (TCEP), and 1 mg/ml lysozyme. Cells were lysed by sonication, and the lysate was cleared by centrifugation for 30 min at 38,000 × *g*, 4 °C. The cleared lysate was mixed with Ni-NTA agarose (Qiagen) and incubated overnight at 4 °C. Ni-NTA beads was transferred into a gravity flow chromatography column and washed with 40 volumes of lysis buffer. The protein was eluted with 125 mM NaCl, 300 mM imidazole, 0.1 mM TCEP, and 25 mM sodium dihydrogenphosphate (pH 7.4). Fractions containing the target protein were pooled and further purified by size exclusion chromatography (HiLoad Superdex 75 16/600 pg, Cytiva, USA) in PBS (pH 7.4); the protein concentration in the pooled eluate fractions was 4.71 mg/ml (307.8 μM) as measured by absorbance at 280 nm. At least 20 mg recombinant α-syn was purified from 1 L of expression culture, and 15 L of culture was purified at a time. Coomassie staining and SEC chromatogram for the protein batch used in the current study, as well as mass spectrometry readout of the first batch produced using this protocol, can be found in the Supplementary Figs. 3 and 4. Prior to long-term storage at −80 °C, the protein was aliquoted at 5 mg per tube (1062 µl) and flash-frozen in liquid nitrogen.

### SAA procedure

The SAA buffer was prepared using 0.1 M PIPES pH 6.5 (BioXtra, Sigma, 80635) with 500 mM NaCl (BioUltra, Sigma, S5150)^54^. PIPES powder was initially diluted in 10 ml of NaOH and filled up to 100 ml (0.111 M) with distilled water, 90 ml of this solution was mixed with 10 ml of 5 M NaCl, and pH was adjusted to 6.5 using NaOH. The buffer was changed from the previously used 0.1 M sodium phosphate pH 7 with 500 NaCl^53^ primarily due to the higher stability and batch-to-batch reproducibility. As one of the Good’s buffers, PIPES is biochemically inert and easy to prepare. We did not otherwise see a relevant difference in the aggregation patterns when comparing these buffers in PD (*n* = 5), MSA (*n* = 3), and control brain lysates (*n* = 3, data not shown, notably the NaCl concentration remained the same and pH nearly the same)^45^. For a full 96-well plate, 10 ml of the buffer mix was prepared. Specifically, 7676 µl of 0.1 M PIPES pH 6.5 with 500 mM NaCl was mixed with 200 µl of 1 mM Thioflavin T (ThT, Biotium, 80033, final concentration 20 µM) and 2124 µl of 4.71 mg/ml recombinant C-terminal his-tagged α-synuclein (final concentration of 1 mg/ml). Six to eight silica glass beads (OPS Diagnostics) were preloaded on the black bottom 96-well plates (Thermo Fisher, 265301). Totally, 95 µl of the buffer mix was added to every well, and finally, 5 µl of the nasal brushing lysate was added to the mixture, resulting in a 1:20 dilution of the sample. Each sample was tested in a technical quadruplicate. On every plate, a positive control (2 µl of 1:100 dilution of a 5% brainstem lysate from a neuropathologically confirmed PD case, Braak Stage 3, Brain Bank Center Würzburg, BrainNet Europe Brain Bank Consortium Network) and a negative control (no sample added) were run in quadruplicate. In the positive control, 3 or 4 replicates had to be positive, and in the negative control, a maximum of 2 positive replicates were allowed for a plate to pass quality control (see Supplementary Fig. 5). The 96-well plates were sealed with a transparent film and incubated for 70 h at 37 °C with cycles of 1 min circular shaking at 432 rpm and 14 min rest with bottom fluorescence readings with a gain of 80 every 45 min in a Tecan Infinite M200 microplate reader (Tecan Group Ltd., Switzerland). Raw data were normalized to a percentage of the maximum fluorescence response (60,000 AU). Values exceeding the maximum fluorescence response (overflow in fluorescence intensity) were capped at 60,000 AU or 100%.

For the spiking experiments, the negative samples (*n* = 50, 91% of all negative samples, as available) were re-tested with and without the addition of the positive control. Namely, to the four replicates, 2 µl of the 1:100 diluted PD brain lysate (see above) was added (i.e., each well contained a total volume of 102 µl: 95 µl SAA buffer, 5 µl of nasal brushing lysate and 2 µl of brain lysate), additional four replicates were run without spiking/addition of brain lysate on the same plate to verify that samples remain negative upon retesting and to allow side-by-side comparison.

### Criteria for sample positivity and SAA parameters

The sample was considered positive, i.e., providing evidence for the presence of misfolded α-syn, if the fluorescence signal of all 4 replicates exceeded the 10% cut-off threshold within 70 h of incubation. If 2 or fewer replicates were positive, the sample was considered negative. If 3 replicates were positive, the sample was retested: if then 4 replicates were positive, the sample was considered positive; if 3 or fewer replicates were positive, then it was deemed negative. Lastly, the other reasons for retesting were a positive result in control samples (*n* = 3) and an unusually short lag phase (*n* = 3). The skin biopsy samples were deemed positive if the fluorescence signal of at least 3 of the 4 replicates reached a 10% cut-off.

The following parameters of SAA were calculated: the averaged maximum ThT fluorescence at the end of 70-h runs referred to as the final percentage of ThT fluorescence (%rfu), and the duration of lag phase (the reaction time (hours) required to cross the 10% fluorescence threshold).

### Hemoglobin ELISA

Hemoglobin concentration in the samples was assessed using a standardized kit (Abcam, ab157707). The samples were tested in 1:20 dilution (the same dilution that is used for SAA) according to the instructions of the manufacturer. Absorbance at 450 nm was measured using Thermo Scientific Multiskan FC. A four-parameter logistic (4PL) regression was used to fit the standards to the sigmoidal curve and interpolate the patient sample concentrations using GraphPad Prism.

### Statistical evaluation

In the previous RT-QuIC/SAA studies of skin biopsies, we saw effect sizes (Cohen’s) between 1.42 and 2.45^53,57^ for double-sided *t*-tests of SAA outcomes (lag times, proportion of positive replicates) and even larger effect sizes (risk ratio) of >6 for Fisher’s exact test based on the proportion of positive subjects in the PD vs. control group. At the time of the planning and ethical approval of this study (2019), no data were available for the sensitivity of pathological α-syn detection in nasal brushings. Based on the limited available data, we based our sample size calculation on the assumption that 10% of control subjects and 50% of PD patients would test positive, which would require a sample size of 25 participants per group (calculated for a power = 0.9, alpha = 0.05, using G*Power software^58^). The post hoc analysis of the actual outcomes revealed a power of 0.93 for the PD vs. control group and 0.96 for the iRBD vs. control group.

For intergroup comparisons of normally distributed data (such as percentage of ThT fluorescence), an unpaired two-sided t-test was performed, and data are reported as mean ± standard deviation. Fisher’s exact test was used for categorical data. Data analysis was performed in R version 4.1.3, ggplot2^59^, and comparegroups^60^ packages were used, and GraphPad Prism Version 9.5.0.

### Reporting summary

Further information on research design is available in the Nature Research Reporting Summary linked to this article.

## Supplementary information

Supplementary Table 2

SUPPLEMENTARY MATERIAL

Reporting Summary

Supplementary dataset (raw SAA data)

## Data Availability

The raw data of SAA assays are included in the Supplementary Dataset. The full detailed demographic and clinical dataset (on a single patient level) is not included to protect patient privacy, but components of it could be made available from the corresponding authors if the request is compliant with directive 95/46/EC and approved by the institutional review board.

## References

[1] Magalhães P, Lashuel HA (2022). Opportunities and challenges of alpha-synuclein as a potential biomarker for Parkinson’s disease and other synucleinopathies. Npj Park. Dis..

[2] Witt M (2009). Biopsies of olfactory epithelium in patients with Parkinson’s disease. Mov. Disord..

[3] Orrú CD (2014). A test for Creutzfeldt-Jakob disease using nasal brushings. N. Engl. J. Med..

[4] Bongianni M (2017). Diagnosis of human prion disease using real-time quaking-induced conversion testing of olfactory mucosa and cerebrospinal fluid samples. JAMA Neurol..

[5] Redaelli V (2017). Detection of prion seeding activity in the olfactory mucosa of patients with fatal familial insomnia. Sci. Rep..

[6] Driver-Dunckley E (2014). Olfactory dysfunction in incidental Lewy body disease and Parkinson’s disease. Parkinsonism Relat. Disord..

[7] Ross GW (2006). Association of olfactory dysfunction with incidental Lewy bodies. Mov. Disord..

[8] Bargar C (2021). Discrimination of MSA-P and MSA-C by RT-QuIC analysis of olfactory mucosa: the first assessment of assay reproducibility between two specialized laboratories. Mol. Neurodegener..

[9] De Luca CMG (2019). Efficient RT-QuIC seeding activity for α-synuclein in olfactory mucosa samples of patients with Parkinson’s disease and multiple system atrophy. Transl. Neurodegener..

[10] Stefani A (2021). Alpha-synuclein seeds in olfactory mucosa of patients with isolated REM sleep behaviour disorder. Brain.

[11] Parkkinen L, Pirttilä T, Alafuzoff I (2008). Applicability of current staging/categorization of alpha-synuclein pathology and their clinical relevance. Acta Neuropathol..

[12] Adler CH (2019). Unified staging system for lewy body disorders: clinicopathologic correlations and comparison to Braak staging. J. Neuropathol. Exp. Neurol..

[13] Kalaitzakis ME, Graeber MB, Gentleman SM, Pearce RKB (2008). The dorsal motor nucleus of the vagus is not an obligatory trigger site of Parkinson’s disease: a critical analysis of α-synuclein staging. Neuropathol. Appl. Neurobiol..

[14] Borghammer P (2021). Neuropathological evidence of body-first vs. brain-first Lewy body disease. Neurobiol. Dis..

[15] Just MK (2022). Alpha-synuclein strain variability in body-first and brain-first synucleinopathies. Front. Aging Neurosci..

[16] Ruf VC (2020). Potential sources of interference with the highly sensitive detection and quantification of alpha-synuclein seeds by qRT-QuIC: Sources of interference with α-Syn qRT-QuIC. FEBS Open Bio.

[17] Cramm M (2016). Stability and Reproducibility Underscore Utility of RT-QuIC for Diagnosis of Creutzfeldt-Jakob Disease. Mol. Neurobiol..

[18] Skrahina V (2021). The Rostock International Parkinson’s Disease (ROPAD) study: protocol and initial findings. Mov. Disord..

[19] Heinzel S (2019). Update of the MDS research criteria for prodromal Parkinson’s disease. Mov. Disord..

[20] Doppler (2014). Cutaneous neuropathy in Parkinson’s disease: a window into brain pathology. Acta Neuropathol..

[21] Wang N, Gibbons CH, Lafo J, Freeman R (2013). α-Synuclein in cutaneous autonomic nerves. Neurology.

[22] Braak H (2003). Staging of brain pathology related to sporadic Parkinson’s disease. Neurobiol. Aging.

[23] Hawkes CH, Del Tredici K, Braak H (2009). Parkinson’s disease: the dual hit theory revisited. Ann. N. Y. Acad. Sci..

[24] Greenland JC, Williams-Gray CH, Barker RA (2019). The clinical heterogeneity of Parkinson’s disease and its therapeutic implications. Eur. J. Neurosci..

[25] Borghammer P (2021). The α-synuclein origin and connectome model (SOC model) of Parkinson’s disease: explaining motor asymmetry, non-motor phenotypes, and cognitive decline. J. Park. Dis..

[26] Berg D (2021). Prodromal Parkinson disease subtypes—key to understanding heterogeneity. Nat. Rev. Neurol..

[27] García-Lorenzo D (2013). The coeruleus/subcoeruleus complex in rapid eye movement sleep behaviour disorders in Parkinson’s disease. Brain.

[28] Shipley MT, Halloran FJ, de la Torre J (1985). Surprisingly rich projection from locus coeruleus to the olfactory bulb in the rat. Brain Res..

[29] Kebschull JM (2016). High-throughput mapping of single-neuron projections by sequencing of barcoded RNA. Neuron.

[30] Doppler K (2022). Association between probable REM sleep behavior disorder and increased dermal alpha-synuclein deposition in Parkinson’s disease. Parkinsonism Relat. Disord..

[31] Postuma RB (2015). REM sleep behavior disorder and neuropathology in Parkinson’s disease. Mov. Disord. J. Mov. Disord. Soc..

[32] Bongianni M (2022). Olfactory swab sampling optimization for α-synuclein aggregate detection in patients with Parkinson’s disease. Transl. Neurodegener..

[33] Tremblay C (2022). Effect of olfactory bulb pathology on olfactory function in normal aging. Brain Pathol..

[34] Nag S (2019). Neocortical Lewy bodies are associated with impaired odor identification in community-dwelling elders without clinical PD. J. Neurol..

[35] Iacono D (2015). Parkinson disease and incidental Lewy body disease: just a question of time?. Neurology.

[36] Parkkinen L (2011). Disentangling the relationship between Lewy bodies and nigral neuronal loss in Parkinson’s disease. J. Park. Dis..

[37] Milber JM (2012). Lewy pathology is not the first sign of degeneration in vulnerable neurons in Parkinson disease. Neurology.

[38] Brann JH, Firestein SJ (2014). A lifetime of neurogenesis in the olfactory system. Front. Neurosci..

[39] Pinna F, Ctenas B, Weber R, Saldiva P, Voegels R (2014). Olfactory neuroepithelium in the superior and middle turbinates: which is the optimal biopsy site?. Int. Arch. Otorhinolaryngol..

[40] Kennel C (2019). Differential expression of mucins in murine olfactory versus respiratory epithelium. Chem. Senses.

[41] Martinez-Anton A (2006). Mucin genes have different expression patterns in healthy and diseased upper airway mucosa. Clin. Htmlent Glyphamp Asciiamp Exp. Allergy.

[42] Davenport KA, Hoover CE, Denkers ND, Mathiason CK, Hoover EA (2018). Modified protein misfolding cyclic amplification overcomes real-time quaking-induced conversion assay inhibitors in deer saliva to detect chronic wasting disease prions. J. Clin. Microbiol..

[43] Shahnawaz M (2020). Discriminating α-synuclein strains in Parkinson’s disease and multiple system atrophy. Nature.

[44] Orrú CD (2011). Prion disease blood test using immunoprecipitation and improved quaking-induced conversion. mBio.

[45] Martinez-Valbuena I (2022). Alpha-synuclein seeding shows a wide heterogeneity in multiple system atrophy. Transl. Neurodegener..

[46] DelleDonne A (2008). Incidental Lewy body disease and preclinical Parkinson disease. Arch. Neurol..

[47] Borghammer P (2022). A postmortem study suggests a revision of the dual-hit hypothesis of Parkinson’s disease. NPJ Park. Dis..

[48] Espay AJ (2017). Biomarker‐driven phenotyping in Parkinson’s disease: a translational missing link in disease‐modifying clinical trials. Mov. Disord..

[49] Postuma RB (2015). MDS clinical diagnostic criteria for Parkinson’s disease: MDS-PD clinical diagnostic criteria. Mov. Disord..

[50] Schenck CH (2013). Rapid eye movement sleep behavior disorder: devising controlled active treatment studies for symptomatic and neuroprotective therapy—a consensus statement from the International Rapid Eye Movement Sleep Behavior Disorder Study Group. Sleep. Med..

[51] Chaudhuri KR (2006). International multicenter pilot study of the first comprehensive self-completed nonmotor symptoms questionnaire for Parkinson’s disease: the NMSQuest study: nonmotor symptoms and PD. Mov. Disord..

[52] Postuma RB (2012). A single-question screen for rapid eye movement sleep behavior disorder: a multicenter validation study: REM sleep behavior disorder screen. Mov. Disord..

[53] Kuzkina A (2021). Diagnostic value of skin RT-QuIC in Parkinson’s disease: a two-laboratory study. Npj Park. Dis..

[54] Concha-Marambio L, Pritzkow S, Shahnawaz M, Farris CM, Soto C (2023). Seed amplification assay for the detection of pathologic alpha-synuclein aggregates in cerebrospinal fluid. Nat. Protoc..

[55] Masuda M (2006). Cysteine misincorporation in bacterially expressed human α-synuclein. FEBS Lett..

[56] Roostaee A, Beaudoin S, Staskevicius A, Roucou X (2013). Aggregation and neurotoxicity of recombinant α-synuclein aggregates initiated by dimerization. Mol. Neurodegener..

[57] Wang Z (2020). Skin α-synuclein aggregation seeding activity as a novel biomarker for Parkinson disease. JAMA Neurol..

[58] Faul F, Erdfelder E, Lang A-G, Buchner A (2007). G*Power 3: a flexible statistical power analysis program for the social, behavioral, and biomedical sciences. Behav. Res. Methods.

[59] Wickham, H. *ggplot2: Elegant Graphics for Data Analysis*. (Springer International Publishing: Imprint: Springer, 2016). 10.1007/978-3-319-24277-4.

[60] Subirana, I., Sanz, H. & Vila, J. Building Bivariate Tables: The compareGroups Package for *R*. *J. Stat. Softw*. **57**, (2014).

