# Supplementary material for: Combining skin and olfactory α-synuclein seed amplification assays (SAA)—towards biomarker-driven phenotyping in synucleinopathies
Source: NPJ Parkinsons Dis. 2023 May 29;9:79. doi: 10.1038/s41531-023-00519-8 (PMC10226020; doi:10.1038/s41531-023-00519-8)
Supplement: Supplementary file 2 — SUPPLEMENTARY MATERIAL [file 41531_2023_519_MOESM2_ESM.pdf]

SUPPLEMENTARY MATERIAL

Combining skin and olfactory α-synuclein seed amplification assays (SAA) - towards biomarker-driven phenotyping in synucleinopathies

Kuzkina A.[1,4*], Rößle J. [1], Seger, A. [2], Panzer C. [1], Kohl A. [1], Maltese V. [1], Musacchio T. [1], Blaschke S.J.[2], Tamgüney G.[9,10], Kaulitz S. [3], Rak K. [3], Scherzad A. [3], Zimmermann P.H.[7,8], Klussmann J.P.[7,8], Hackenberg S. [3,5], Volkmann J. [1], Sommer C. [1], Sommerauer M. [2, 6], Doppler K. [1*]

Address correspondence to: Anastasia Kuzkina at akuzkina@bwh.harvard.edu and Kathrin Doppler at doppler_k@ukw.de

CONTENTS:

Supplementary Figure 1 Serial dilutions of PD and control samples
Supplementary Figure 2 Additional examples of pronounced nasal brushing matrix interference with the SAA.
Supplementary Figure 3 Purification of recombinant α-synuclein (SAA substrate)
Supplementary Figure 4 Mass spectrometry analysis of the substrate
Supplementary Figure 5 Technical controls
Supplementary Table 1 Characteristics of patients with a negative skin biopsy compared to the rest of the PD cohort.
Supplementary Figure 6 All SAA graphs of the dataset
Supplementary Dataset: All raw SAA data – see separate excel file.

Supplementary Figure 1: Serial dilutions of PD and control samples. **A)** Distribution of the number of positive replicates (0-4) at different sample dilutions (1:20 – 1:1000) in the control (n=5) and PD (n=5) samples. **B)** SAA curves of PD samples with different seeding capacities. **C)** SAA curves of two of the control samples.

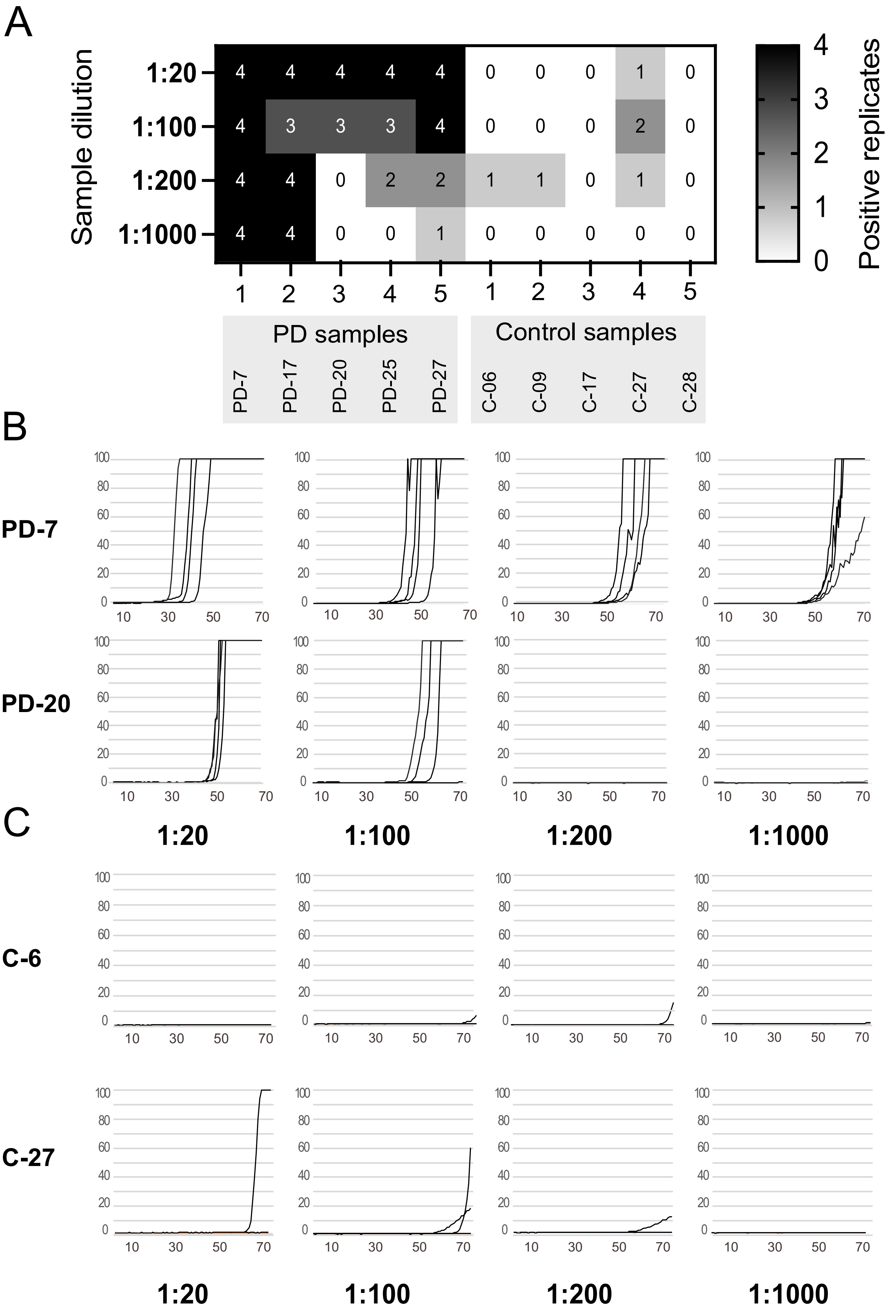

Supplementary Figure 2: Additional examples of pronounced interference of nasal brushing matrix with the a-syn aggregation tested by the addition of a known seed (PD brain lysate) **A)** The majority of the tested negative samples (n=38, 76%) showed a prolongation of the lag phase to varying degrees, two examples are shown here. **B)** Some samples, n=12 (24%), could inhibit a-syn aggregation to the extent that the outcome was deemed negative. Two such examples (both replicated twice) are shown. The proportion of samples inhibiting the SAA was similar in different disease groups (6 out of 27 (22%) for controls, 3 of 14 (21%) for PD, and 3 out of 9 (33%) for iRBD). C) Examples of the other four samples (5 in total, or 10% of tested negative samples) that changed the ThT curve morphology of the PD brain lysate.

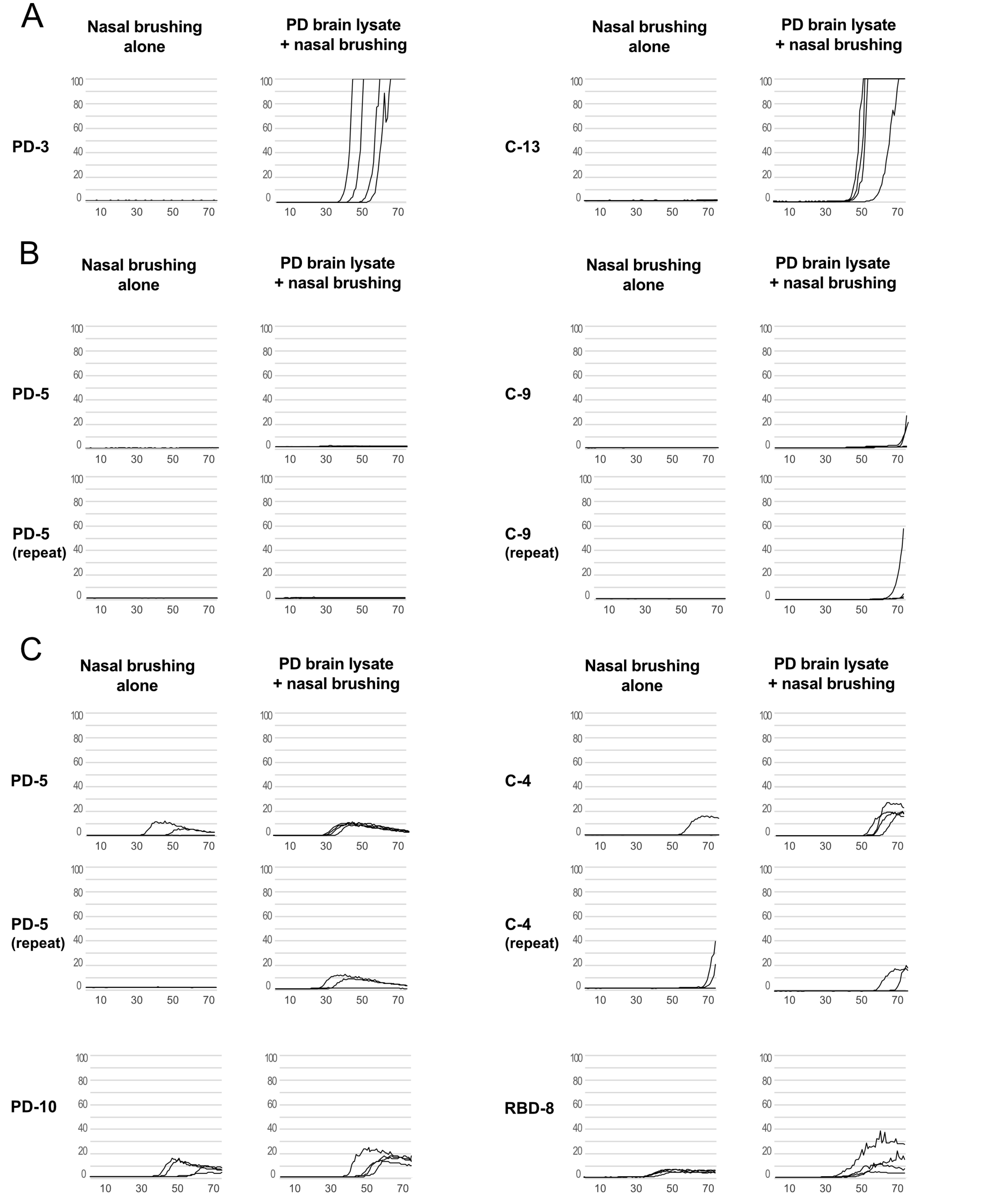

Supplementary Figure 3 Purification of recombinant α-synuclein (SAA substrate). E. coli strain BL21 (DE3) was transformed with plasmid pET21-b-α-syn (Y136TAT). **A)** Coomassie stain of an SDS-PAGE of different purification steps. WCE: whole cell lysate. SL: soluble lysate. FT: flow-through. # size exclusion chromatography (SEC) eluates. **B)** Merged SEC chromatograms (IMAC pool was loaded on the column in two consecutive runs). Eluates #D2-D8 were pooled.

A

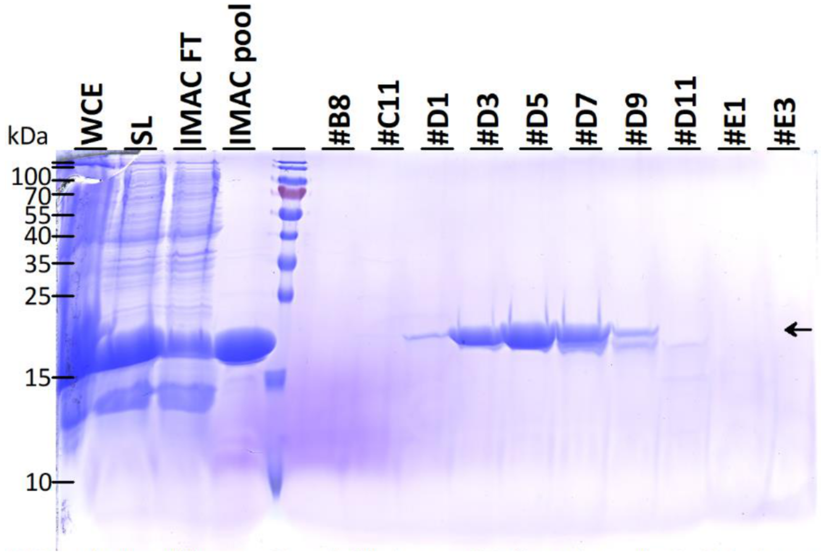

B

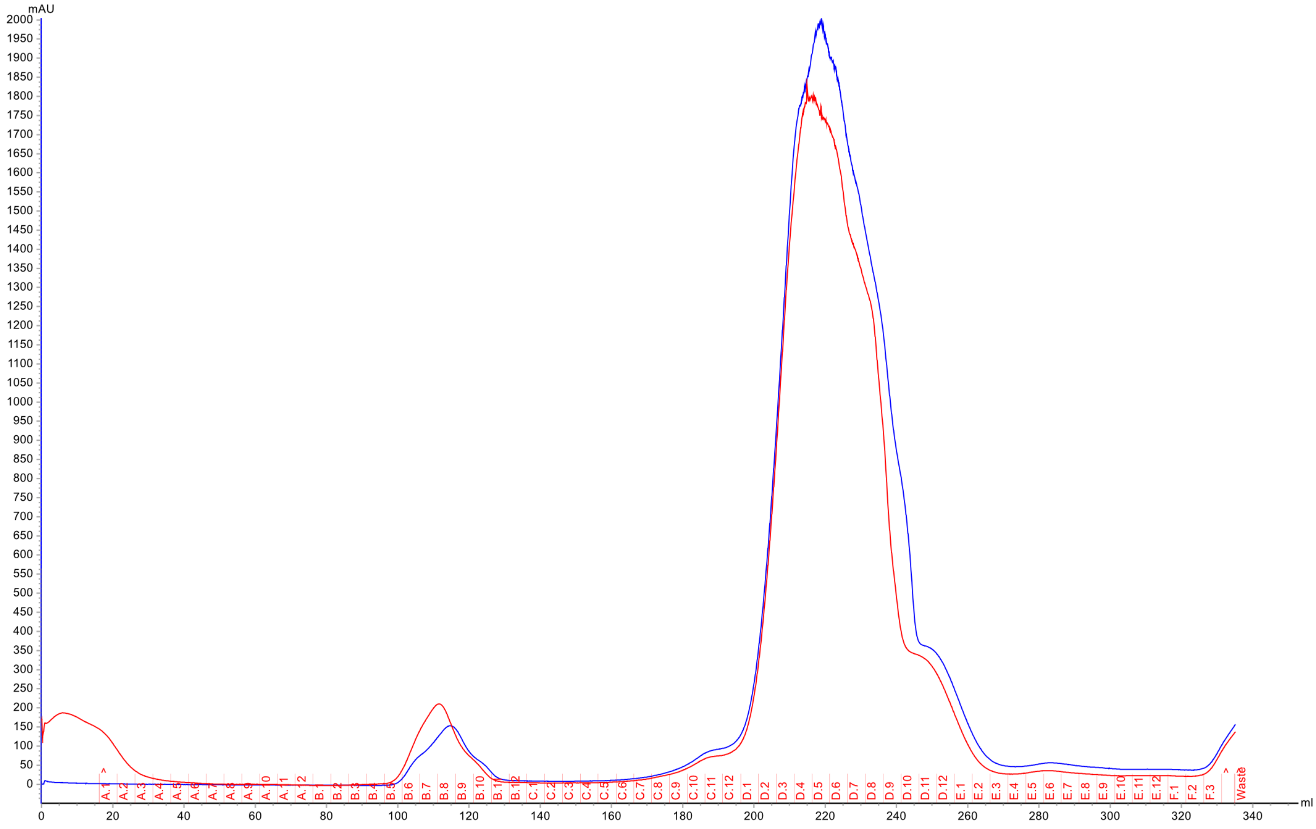

Supplementary Figure 4 Mass spectrometry analysis of the substrate **A)** SDS-PAGE with Coomassie staining of the analyte prior to trypsin digestion. **B)** Custom database (AGMS) search using the complete protein sequence including the C-terminal 6xhistidine-tag showed 100% sequence coverage. **C)** SwissProt database search: matches with α-synuclein and exponentially modified protein abundance indexes (emPAI) are shaded green.

A

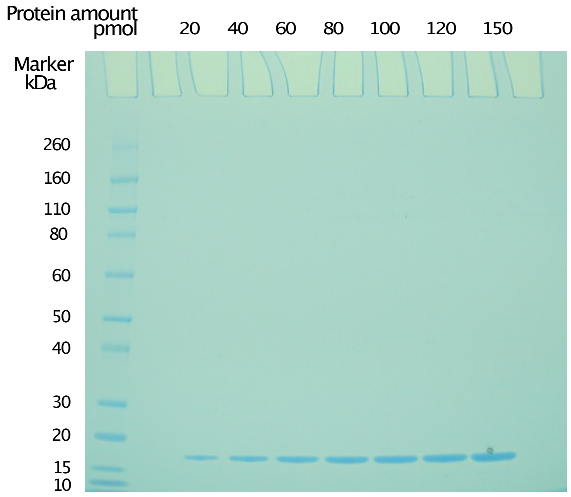

B

Protein View: agms:531_alpha-syn_TAT_hispro

alpha-syn_TAT_his.pro

**Database:**	agms
**Score:**	7901
**Nominal mass (M_r):**	15274
**Calculated pI:**	5.22

Sequence similarity is available as an NCBI BLAST search of agms:531_alpha-syn_TAT_hispro against

Search parameters

**Enzyme:**	Trypsin: cuts C-term side of KR unless next residue is P.
**Fixed modifications:**	Carbamidomethyl (C)
**Variable modifications:**	Acetyl (Protein N-term), Gln->pyro-Glu (N-term Q), Oxidation (M)

Protein sequence coverage: 100%

Matched peptides shown in ***bold red***.

```
  1 MDVFMKGLSK AKEGVVAAAE KTKQGVAEAA GKTKEGVLYV GSKTKEGVVH
 51 GVATVAEKTK EQVTNVGGAV VTGVTAVAQK TVEGAGSIAA ATGFVKKDQL
101 GKNEEGAPQE GILEDMPVDP DNEAYEMPSE EGYQDYEPEA HHHHHH
```

C

Family	Member	Accession	Score	Mass	Num. of matches	Num. of significant matches	Num. of sequences	Num. of significant sequences	emPAI	Description
1	1	sp|P37840-2|SYUA_HUMAN	5476	11365	175	150	28	28	55544.44	Isoform 2-4of Alpha-synuclein OS=Homo sapiens OX=9606 GN=SNCA
1	2	sp|P61142|SYUA_MACFA	3865	14464	151	126	27	27	4435.21	Alpha-synuclein OS=Macaca fascicularis OX=9541 GN=SNCA PE=3 SV=1
1	3	sp|O55042-2|SYUA_MOUSE	3087	12395	117	105	23	23	6718.85	Isoform 2 of Alpha-synuclein OS=Mus musculus OX=10090 GN=Snca
1	4	sp|Q3T0G8|SYUA_BOVIN	1905	14499	90	78	16	16	258.07	Alpha-synuclein OS=Bos taurus OX=9913 GN=SNCA PE=2 SV=1
1	5	sp|Q91448|SYUA_SERCA	1431	14864	88	76	16	16	293.1	Alpha-synuclein OS=Serinus canaria OX=9135 GN=SNCA PE=2 SV=1
2	1	sp|C0PZP0|RL13_SALPC	1884	16009	43	43	18	18	251.61	50S ribosomal protein L13 OS=Salmonella paratyphi C (strain RKS4594) OX=476213 GN=rplM PE=3 SV=1
3	1	sp|P0A9A9|FUR_ECOLI	1172	17012	23	23	13	13	31.37	Ferric uptake regulation protein OS=Escherichia coli (strain K12) OX=83333 GN=fur PE=1 SV=1
4	1	sp|P0A7W1|RS5_ECOLI	336	17592	8	7	7	6	2.54	30S ribosomal protein S5 OS=Escherichia coli (strain K12) OX=83333 GN=rpsE PE=1 SV=2
5	1	sp|P00761|TRYP_PIG	327	25078	8	8	5	5	1.44	Trypsin OS=Sus scrofa OX=9823 PE=1 SV=1
6	1	sp|C0Q0C4|RS12_SALPC	188	13957	9	7	9	7	5.27	30S ribosomal protein S12 OS=Salmonella paratyphi C (strain RKS4594) OX=476213 GN=rpsL PE=3 SV=1
8	1	sp|P0AFX4|RSD_ECOLI	128	18288	3	3	3	3	0.83	Regulator of sigma D OS=Escherichia coli (strain K12) OX=83333 GN=rsd PE=1 SV=1
10	1	sp|P0A7M2|RL28_ECOLI	89	9058	3	3	3	3	2.28	50S ribosomal protein L28 OS=Escherichia coli (strain K12) OX=83333 GN=rpmB PE=1 SV=2

Supplementary Figure 5 Technical controls. Raw data of the positive control (lysate of the midbrain of a Braak stage 3 PD) and negative control (unseeded substrate in SAA buffer, i.e. recombinant protein alone). **A)** Left: Lag times of 15 separate experiments (from all plates on which the samples included in the current study were run) of the positive and negative controls. Mean ±SD are shown in gray lines. Right: number of positive replicates (crossing 10% rfu within 70 hours) in the same n=15 runs. **B)** ThT curves of the experiments. Individual means and S.E.M are plotted. **C)** Mean values of all tests ±S.E.M. for the positive and negative control are plotted. **D)** Individual plots of all negative controls used in the study (n=15) **E)** Individual plots of the corresponding positive controls (n=15).

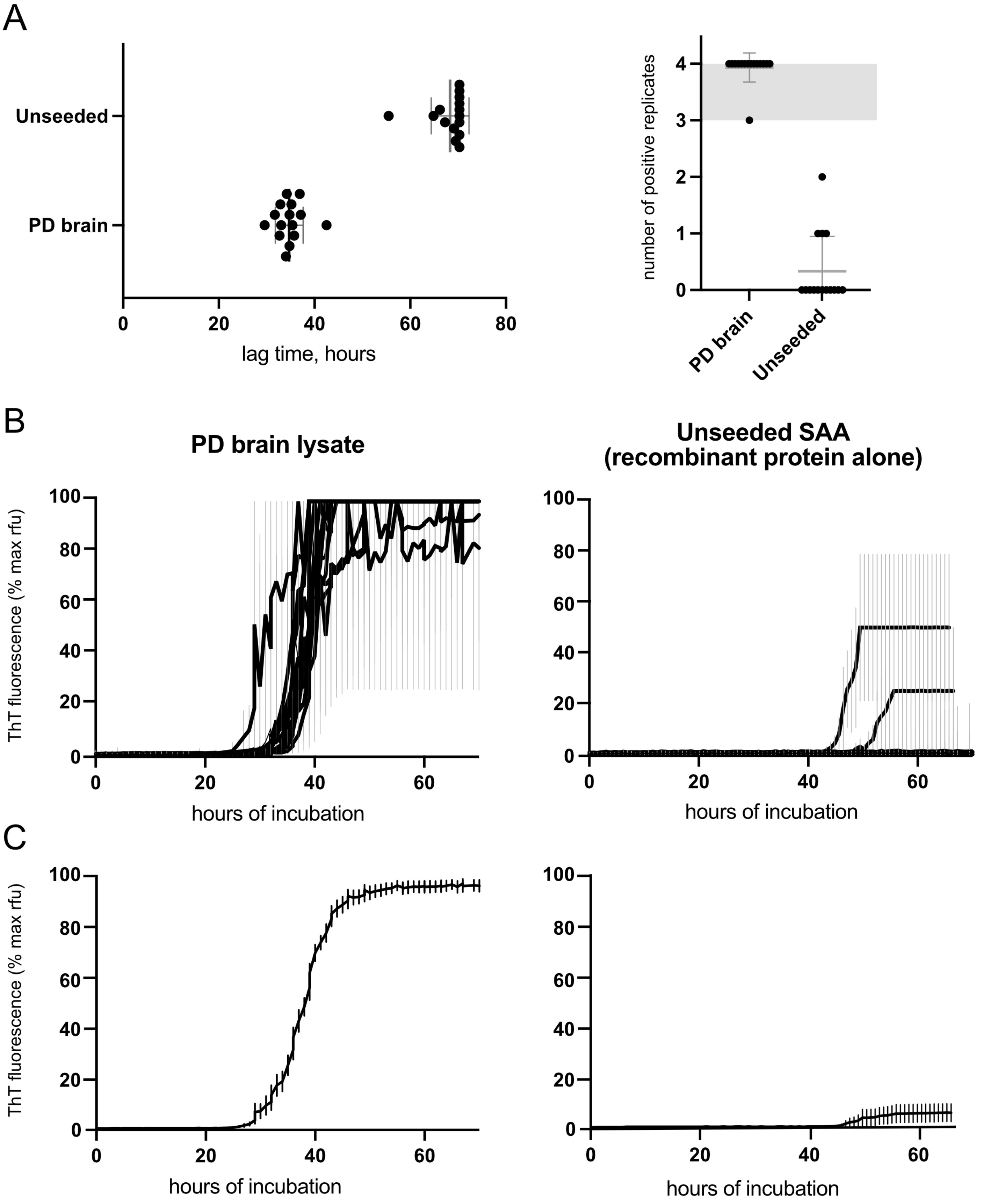

D

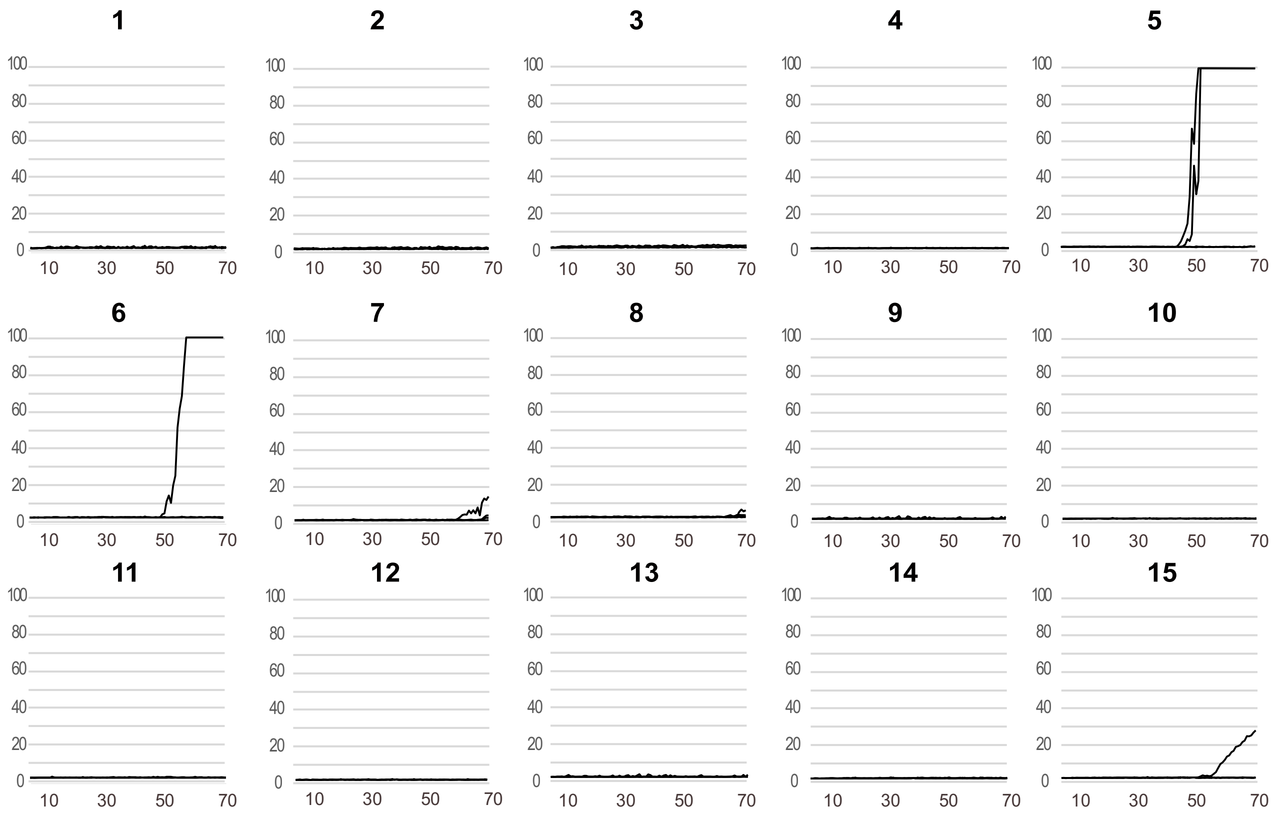
1
2
3
4
5
6
7
8
9
10
11
12
13
14
15
0 20 40 60 80 100
10 30 50 70

E

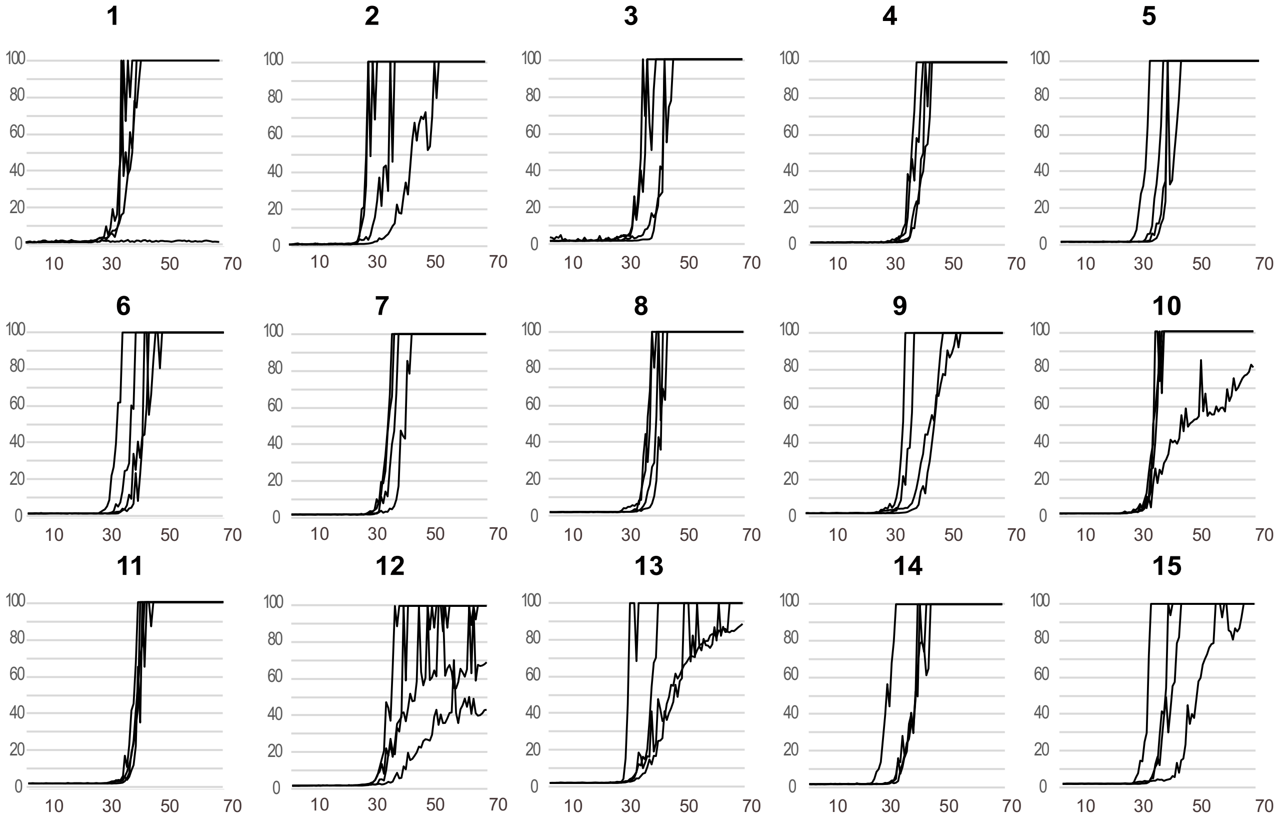
1
2
3
4
5
6
7
8
9
10
11
12
13
14
15
0 20 40 60 80 100
10 30 50 70

Supplementary Table 1: Characteristics of patients with a negative skin biopsy compared to the rest of the PD cohort.

<table>
<tr><th colspan="2"></th><th>PD patients with a negative skin SAA (presumed brain-first), N=4*</th><th>The rest of the PD cohort (who received both tests), N=15</th><th>p-value</th></tr>
<tr><td colspan="2">female:male ratio</td><td>1:3</td><td>5:10</td><td>1</td></tr>
<tr><td colspan="2">MoCA, average (SD)</td><td>27.8 (1.71)</td><td>27.5 (2.00)</td><td>0.836</td></tr>
<tr><td colspan="2">MCI, n (%)</td><td>0 (0.00%)</td><td>2 (13.3%)</td><td>1</td></tr>
<tr><td colspan="2">Motor subtype:</td><td colspan="2"></td><td rowspan="4">0.361</td></tr>
<tr><td colspan="2">akinetic-rigid, n (%)</td><td>1 (25.0%)</td><td>10 (66.7%)</td></tr>
<tr><td colspan="2">mixed, n (%)</td><td>2 (50.0%)</td><td>4 (26.7%)</td></tr>
<tr><td colspan="2">tremor-dominant, n (%)</td><td>1 (25.0%)</td><td>1 (6.67%)</td></tr>
<tr><td colspan="2">Sniffin' sticks, sum (SD)</td><td>21.6 (8.92)</td><td>18.6 (5.58)</td><td>0.574</td></tr>
<tr><td colspan="2">H&Y stage, average (SD)</td><td>2.00 (0.00)</td><td>2.47 (0.52)</td><td>0.004</td></tr>
<tr><td colspan="2">Disease duration, average (SD), excluding a single patient with 1-year duration</td><td>7.00 (4.97), 9 (3.6)</td><td>13.9 (4.39)</td><td>0.061</td></tr>
<tr><td colspan="2">Age at diagnosis, average (SD)</td><td>56.8 (14.1)</td><td>49.7 (10.3)</td><td>0.406</td></tr>
<tr><td colspan="2">Familial PD history, n (%)</td><td>0 (0.00%)</td><td>5 (33.3%)</td><td>0.53</td></tr>
<tr><td colspan="2">DBS surgery, n (%)</td><td>3 (75.0%)</td><td>10 (66.7%)</td><td>1</td></tr>
<tr><td colspan="2">positive RBD screening, n (%)</td><td>0 (0.00%)</td><td>9 (60.0%)</td><td>0.087</td></tr>
<tr><td colspan="2">NMSQ sum, average (SD)</td><td>6.00 (1.41)</td><td>9.93 (4.53)</td><td>0.011</td></tr>
<tr><td colspan="5">Responses in NMSQ with most striking differences</td></tr>
<tr><td rowspan="8">NMSQ questionnaire (Number of patients who reported the symptom)</td><td>5. Constipation (less than 3 bowel movements a week) or having to strain to pass a stool (faeces)</td><td>0 (0.00%)</td><td colspan="2">6 (40.0%)</td></tr>
<tr><td>11. Unexplained change in weight (not due to change in diet)</td><td>0 (0.00%)</td><td colspan="2">5 (33.3%)</td></tr>
<tr><td>12. Problems remembering things that have happened recently or forgetting to do things</td><td>0 (0.00%)</td><td colspan="2">7 (46.7%)</td></tr>
<tr><td>14. Seeing or hearing things that you know or are told are not there</td><td>0 (0.00%)</td><td colspan="2">5 (33.3%)</td></tr>
<tr><td>20. Feeling lightheaded, dizzy or weak standing from sitting or lying</td><td>0 (0.00%)</td><td colspan="2">4 (26.7%)</td></tr>
<tr><td>21. Falling</td><td>0 (0.00%)</td><td colspan="2">3 (20.0%)</td></tr>
<tr><td>22. Finding it difficult to stay awake during activities such as working, driving or eating</td><td>0 (0.00%)</td><td colspan="2">5 (33.3%)</td></tr>
<tr><td>24. Intense, vivid dreams or frightening dreams</td><td>0 (0.00%)</td><td colspan="2">8 (53.3%)</td></tr>
</table>

	25. Talking or moving about in your sleep as if you are ‘acting’ out a dream	0 (0.00%)	8 (53.3%)	
	29. Double vision	0 (0.00%)	4 (26.7%)	
UPDRS part 1, sum (SD)		3.75 (2.63)	6.67 (5.02)	0.146
UPDRS part 2, sum (SD)		8.50 (6.76)	14.5 (4.76)	0.172
UPDRS part 4, sum (SD)		2.25 (4.50)	5.60 (5.05)	0.252
**Responses in MDS-UPDRS with most striking differences**				
**MDS-UPDRS scale. Average response for the group. Each symptom's intensity/frequency was rated from 0 to 4**	1.11 Over the past week have you had constipation troubles that cause you difficulty moving your bowels?	0.00 (0.00)	1.13 (1.06)	0.001
	1.12 Over the past week, have you felt faint, dizzy, or foggy when you stand up after sitting or lying down?	0.00 (0.00)	0.60 (0.99)	0.033
	2.8 Over the past week, have you usually had trouble doing your hobbies or other things that you like to do?	0.25 (0.50)	1.47 (1.30)	0.012
	2.12 Over the past week, have you usually had problems with balance and walking?	0.25 (0.50)	1.60 (1.12)	0.004
	2.13 Over the past week, on your usual day when walking, do you suddenly stop or freeze as if your feet are stuck to the floor?	0.00 (0.00)	0.80 (1.08)	0.013
	4.1 Time spent with dyskinesias	0.00 (0.00)	0.60 (0.83)	0.014
	4.2 Functional impact of dyskinesias	0.00 (0.00)	0.87 (1.51)	0.043

* Nasal brushing SAA was positive in 3 of these patients; in the remaining patient (PD-5), nasal brushing was negative and also found to inhibit aggregation of the positive control (i.e. false-negative is possible).

Parkinson's disease

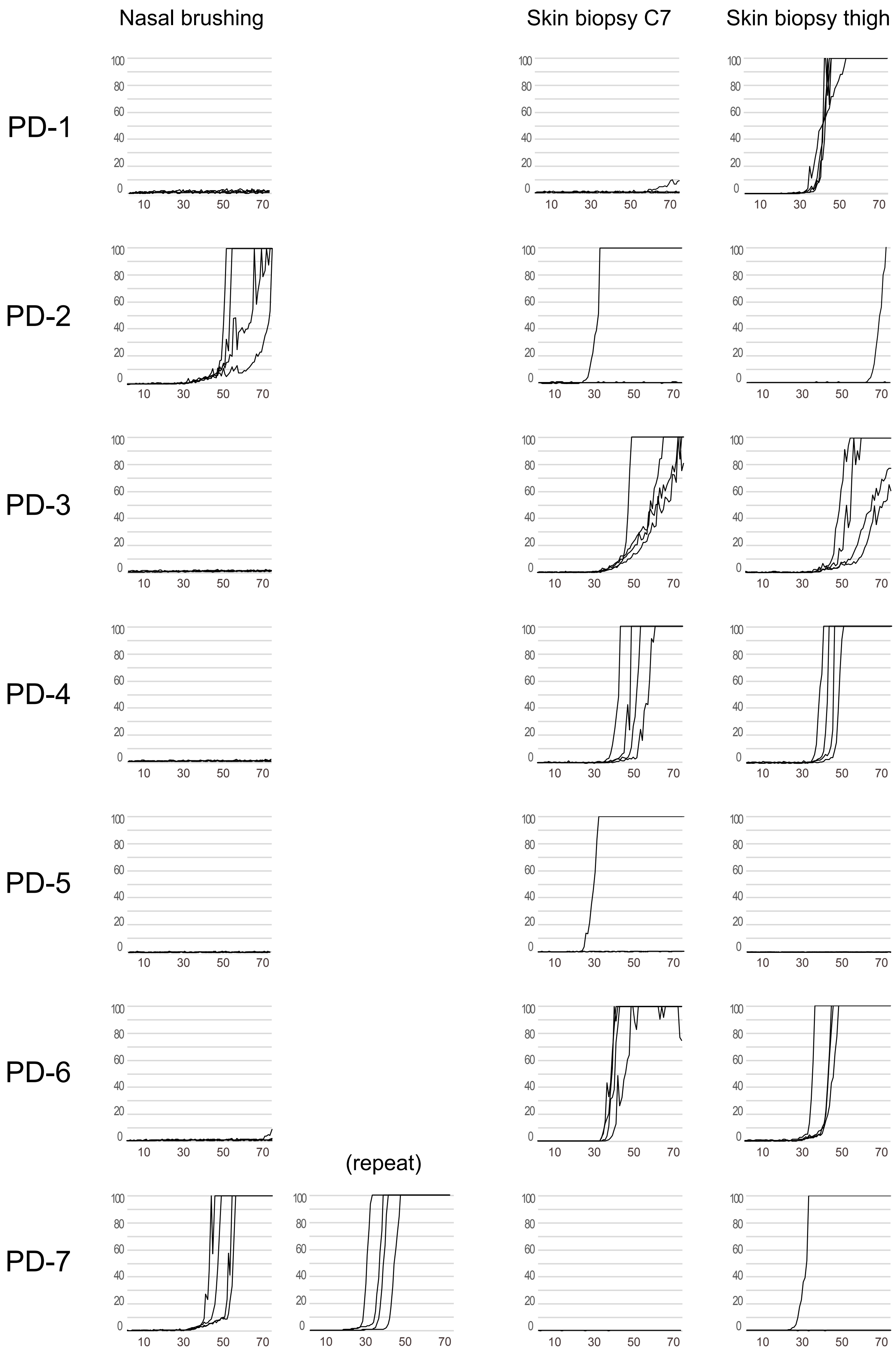

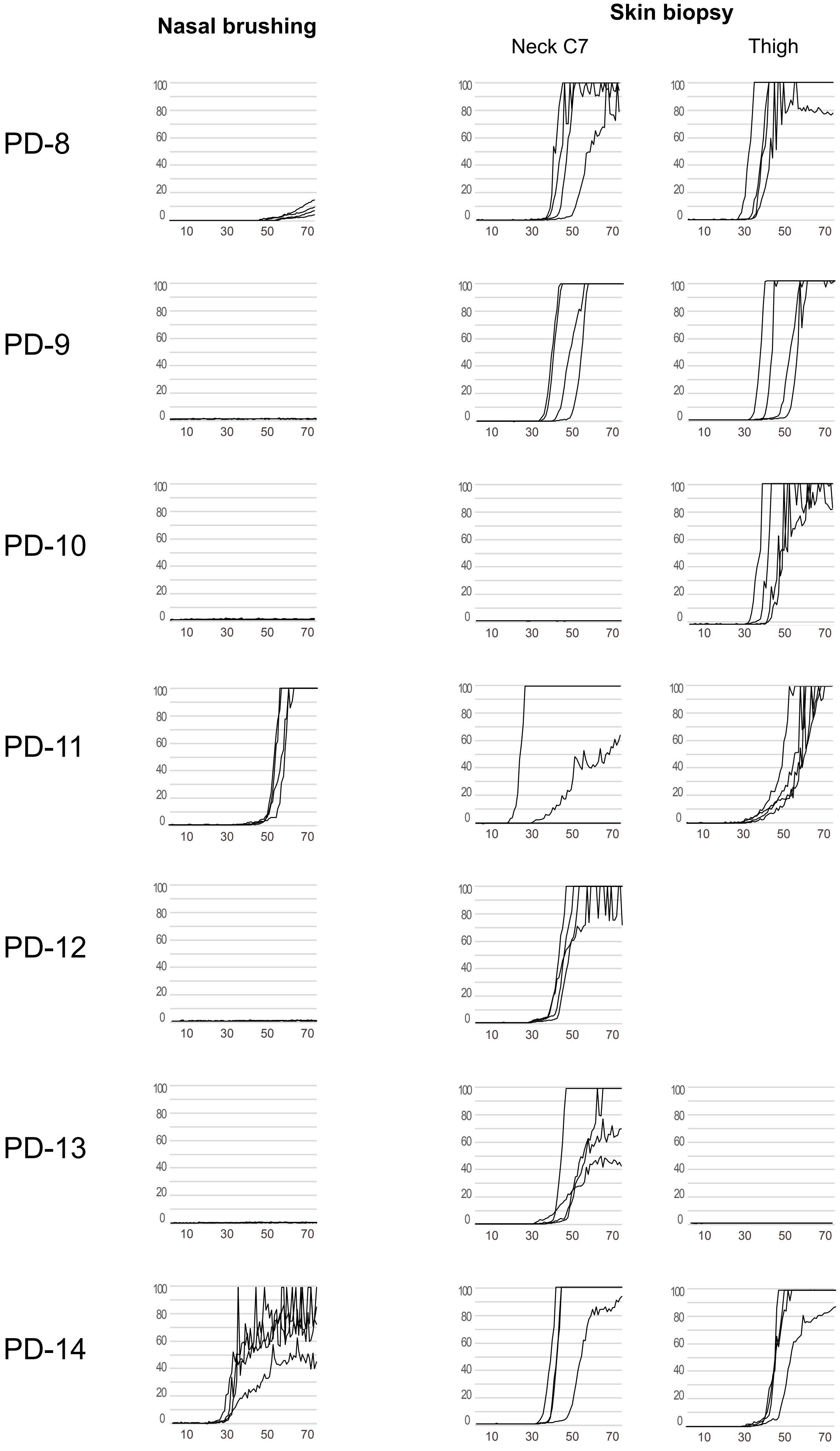
Nasal brushing
Skin biopsy
Neck C7
Thigh
PD-8
PD-9
PD-10
PD-11
PD-12
PD-13
PD-14
0
20
40
60
80
100
10
30
50
70

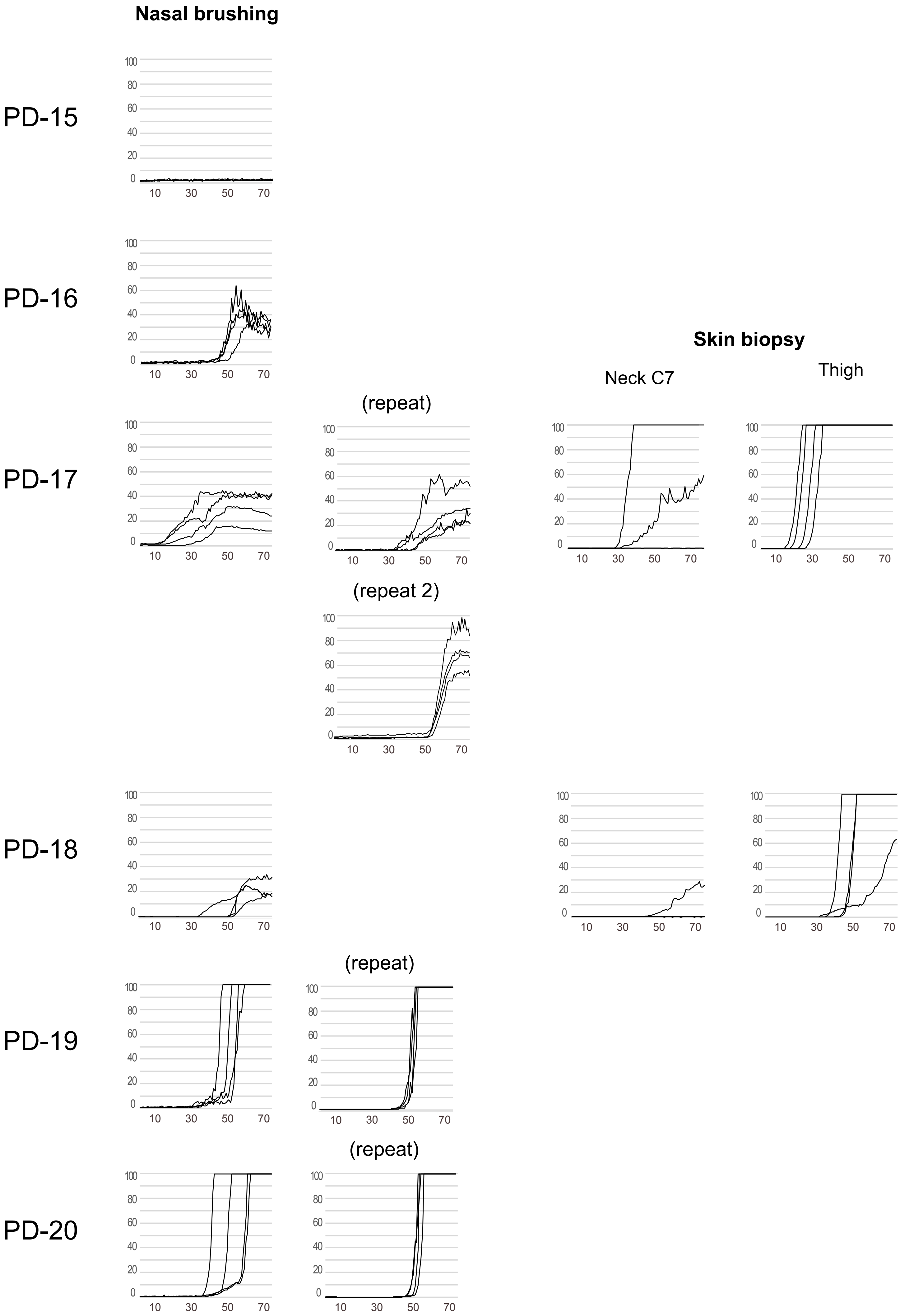
Nasal brushing
Skin biopsy
Neck C7
Thigh
PD-15
PD-16
PD-17
(repeat)
(repeat 2)
PD-18
PD-19
(repeat)
PD-20
(repeat)
0
20
40
60
80
100
10
30
50
70

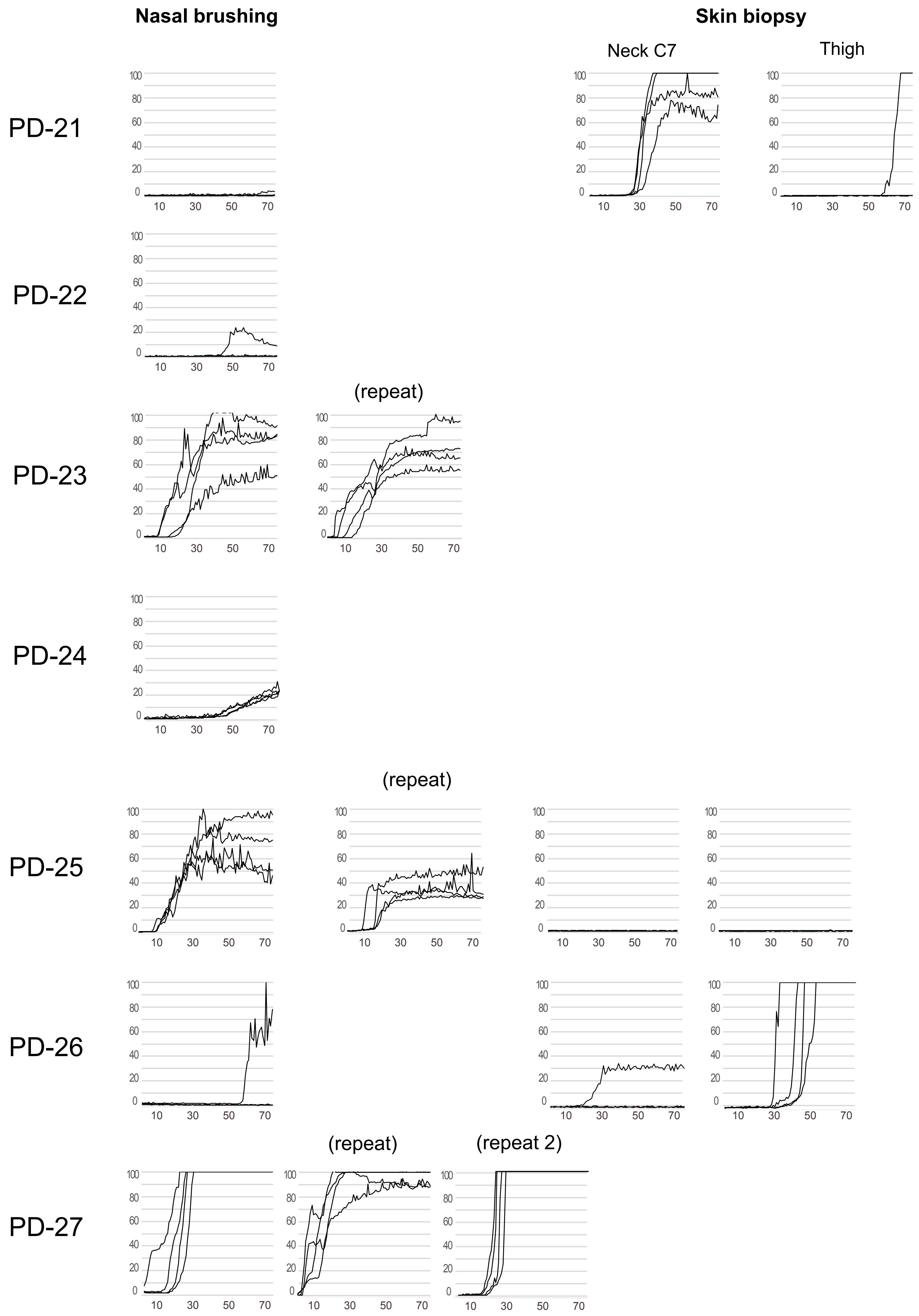
Nasal brushing
Skin biopsy
Neck C7
Thigh
PD-21
PD-22
(repeat)
PD-23
PD-24
(repeat)
PD-25
PD-26
(repeat)
(repeat 2)
PD-27
0
20
40
60
80
100
10
30
50
70

REM sleep behavior disorder

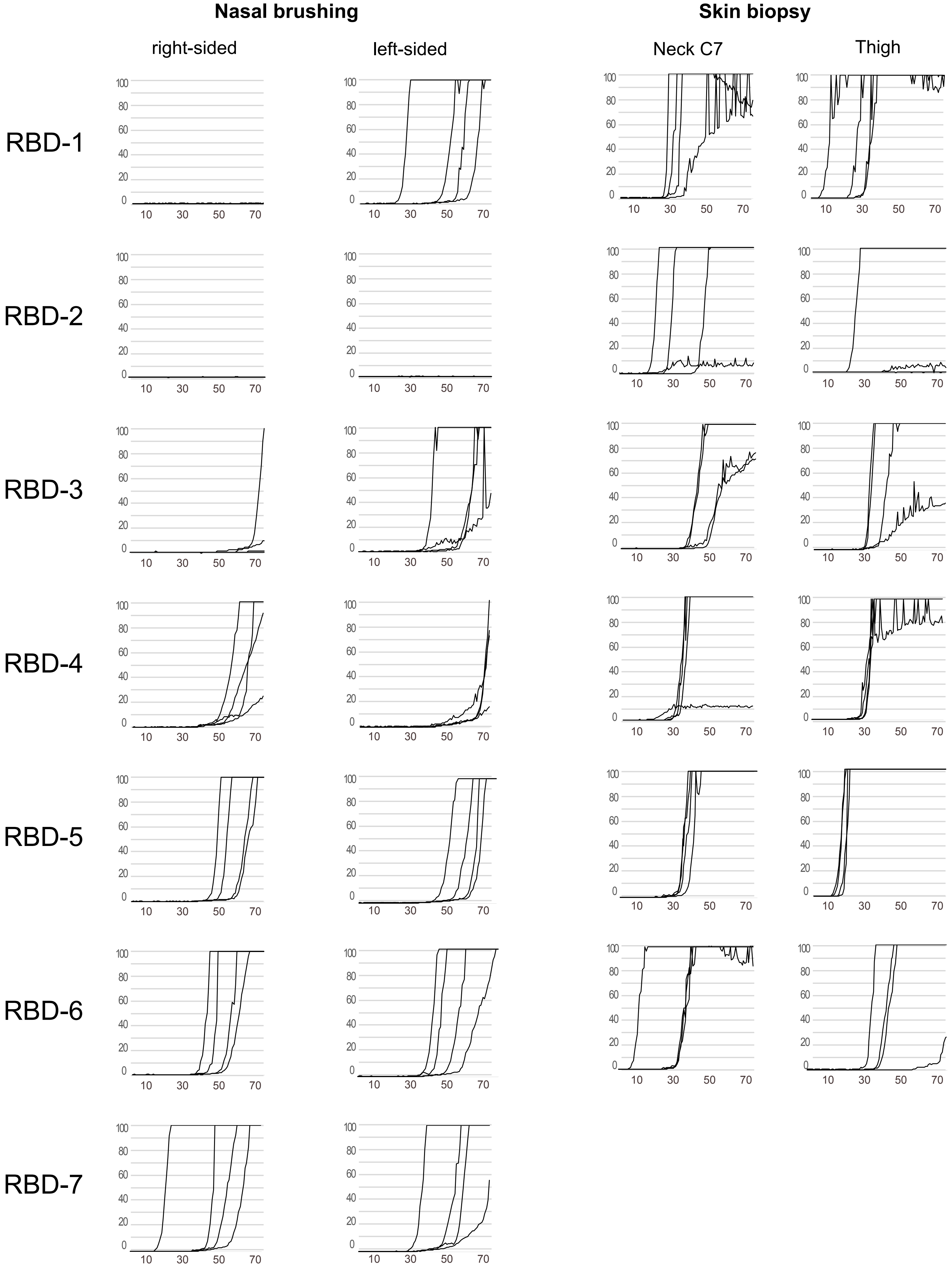

REM sleep behavior disorder

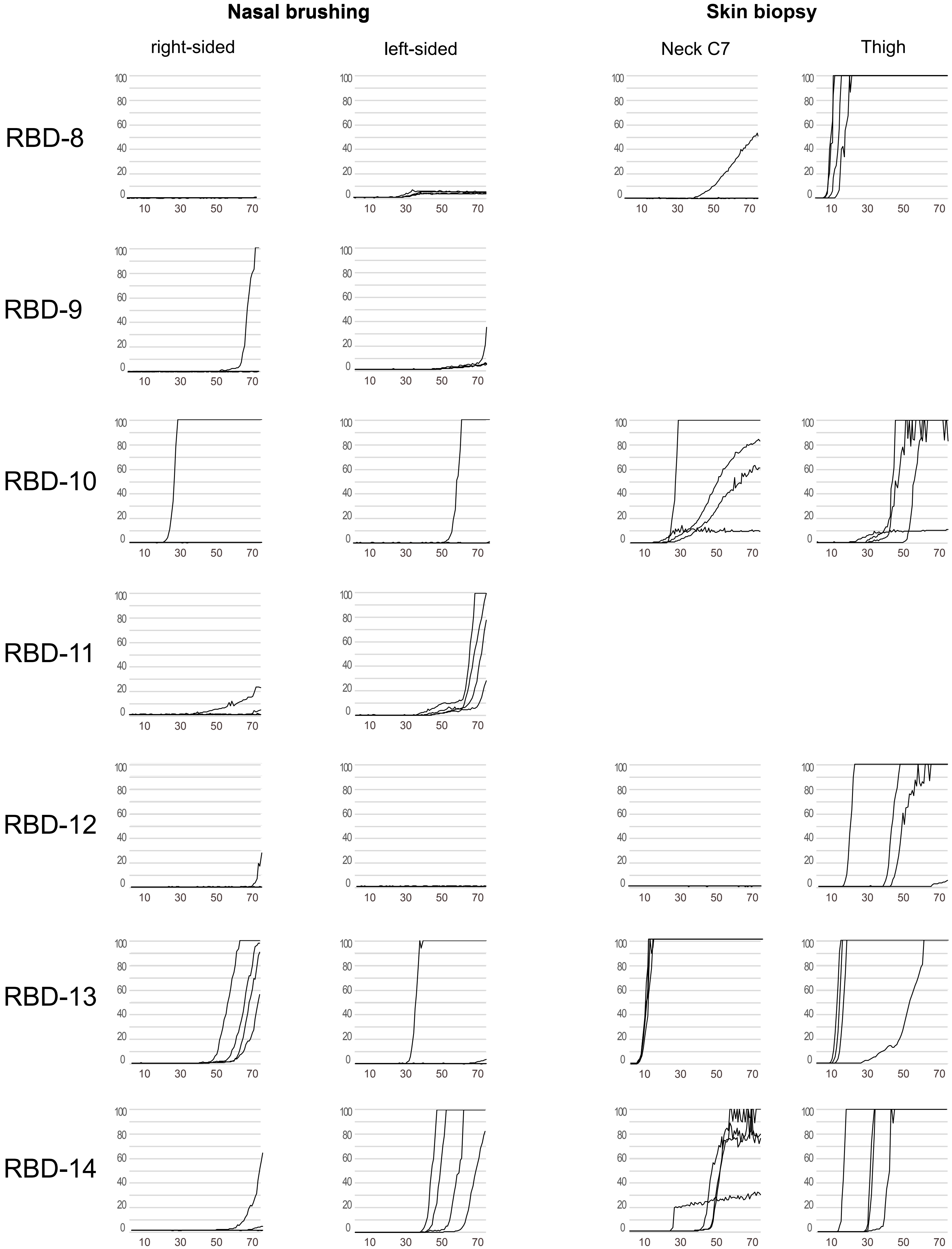

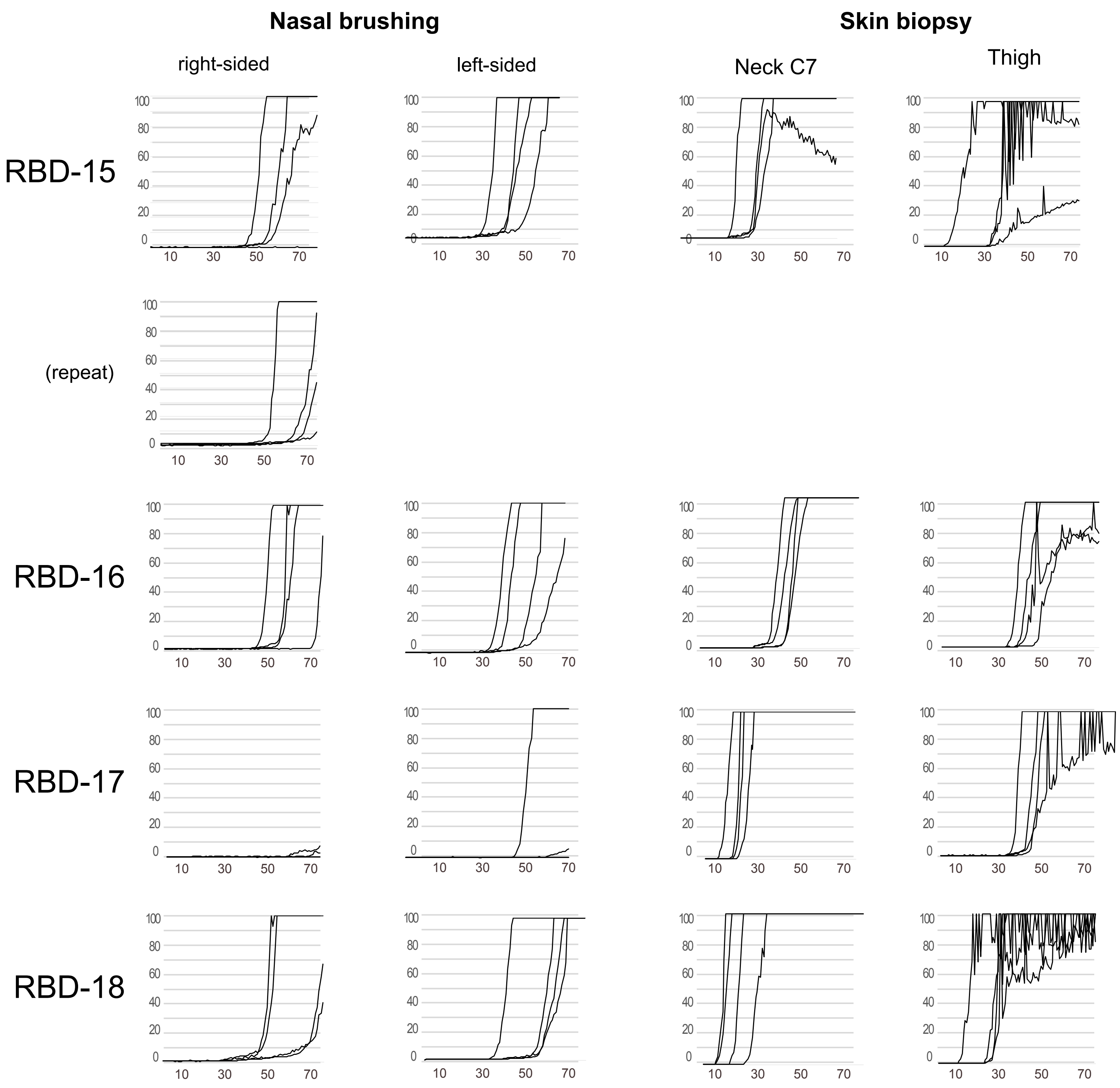
Nasal brushing
Skin biopsy
right-sided
left-sided
Neck C7
Thigh
RBD-15
(repeat)
RBD-16
RBD-17
RBD-18

Control group

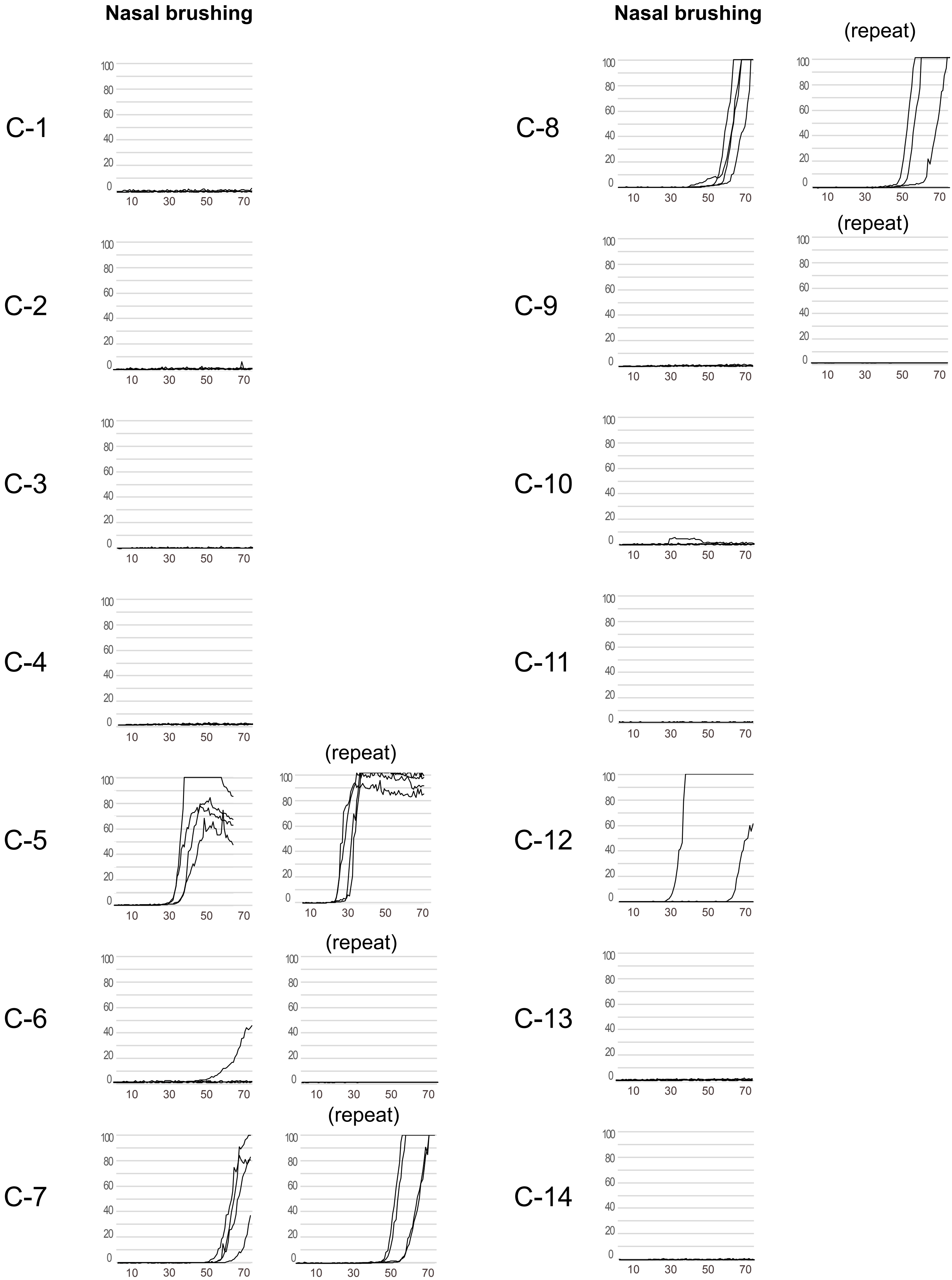

Control group

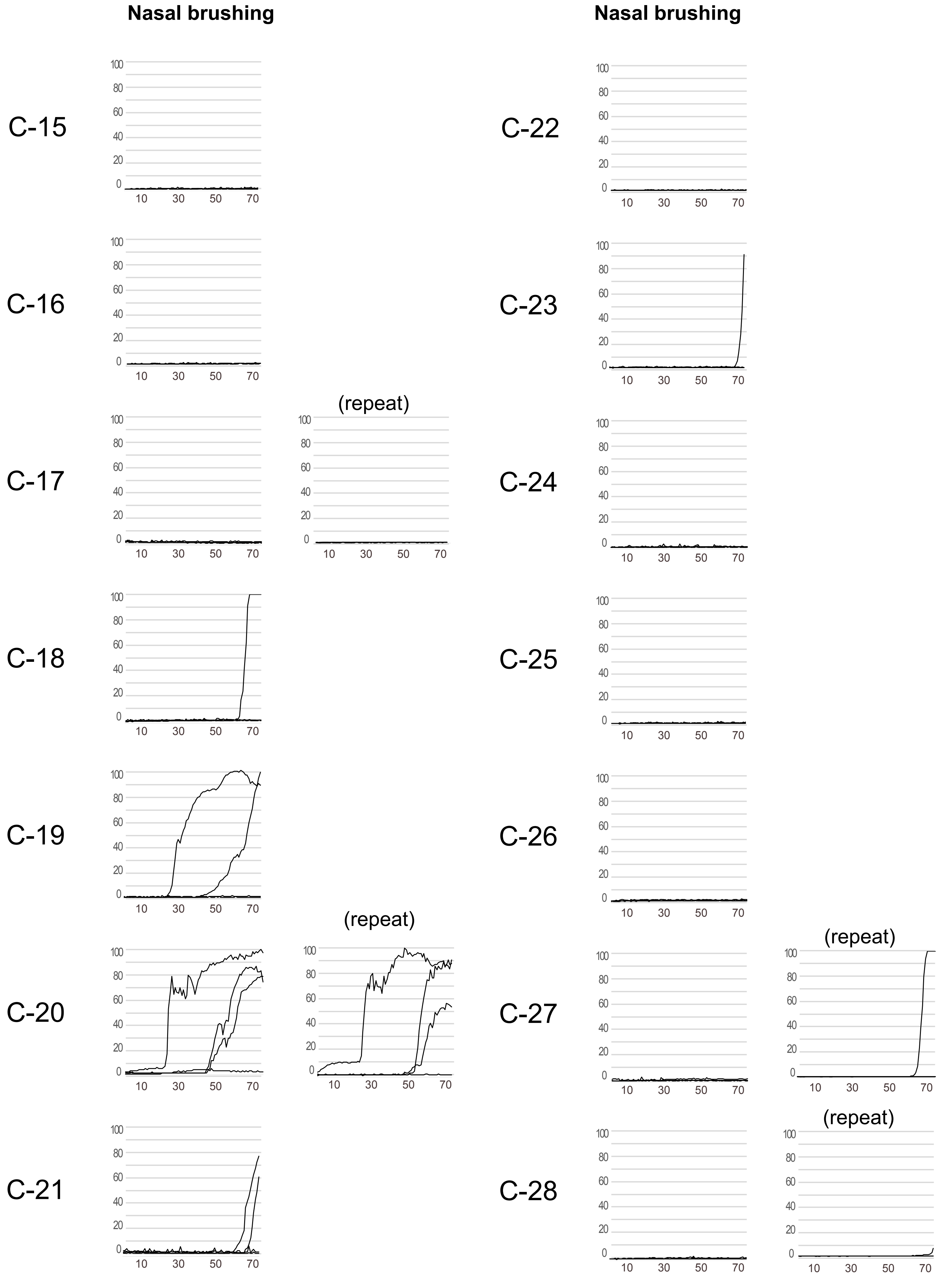

Control group

Nasal brushing

C-29

C-30

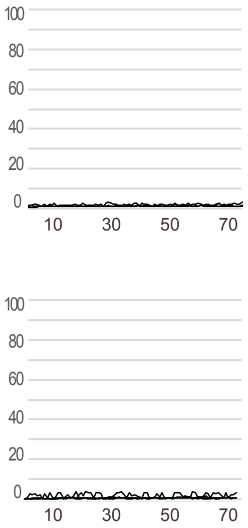

MSA

Nasal brushing

Skin biopsy

Neck C7

Thigh

MSA-1
(MSA-P)

MSA-2
(MSA-C)

MSA-3
(MSA-P)

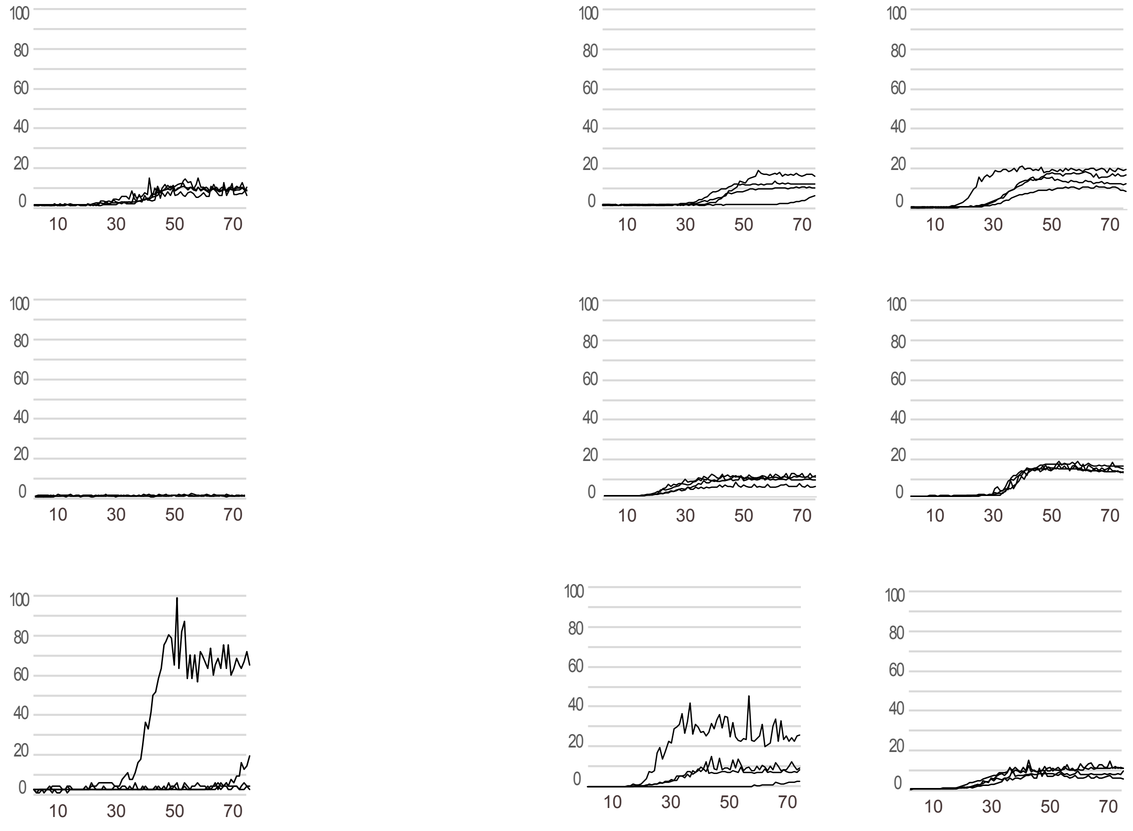

PSP

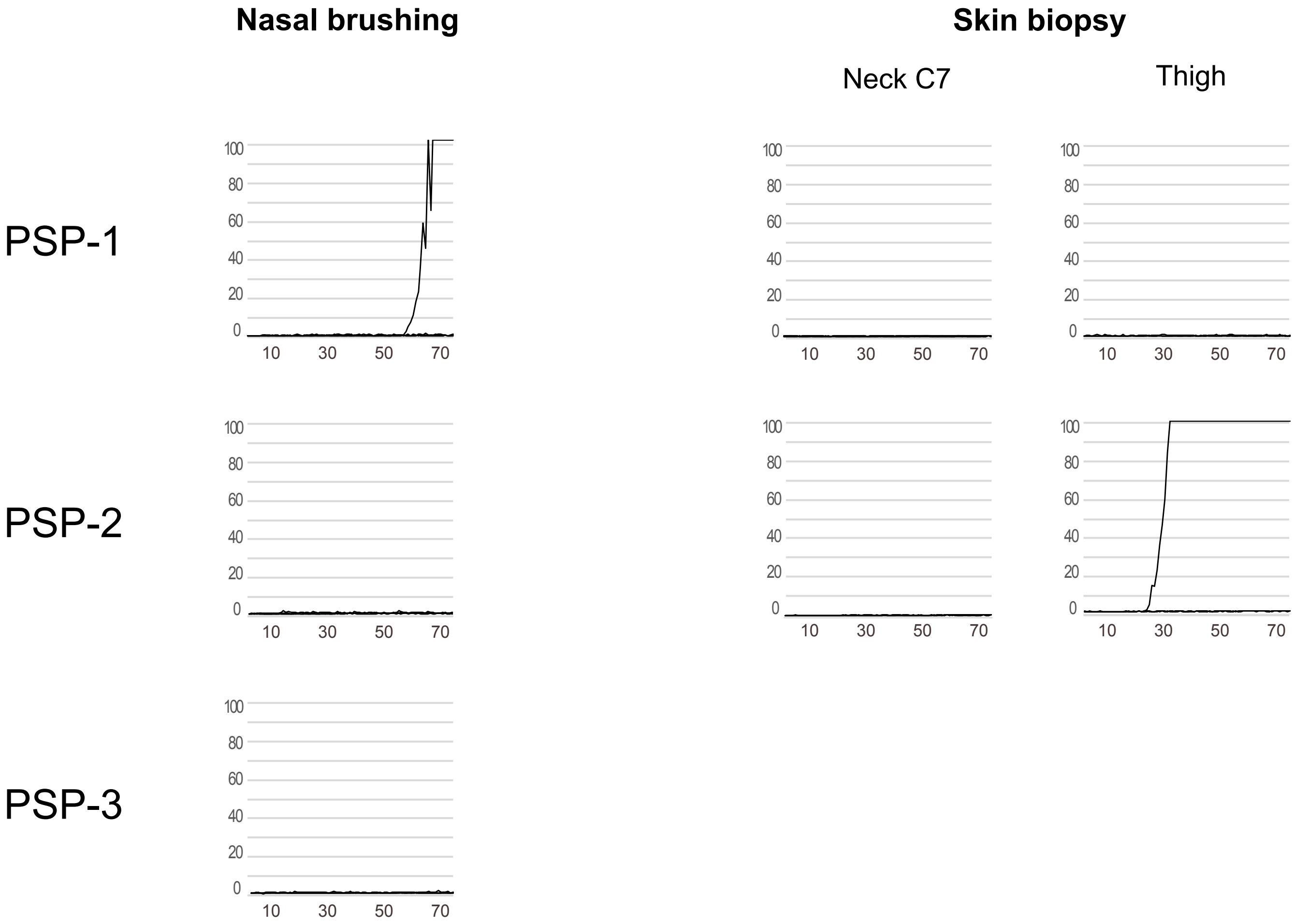

Nasal brushing
Skin biopsy
Neck C7
Thigh
PSP-1
PSP-2
PSP-3
0
20
40
60
80
100
10
30
50
70
