# Supplementary material for: Combining skin and olfactory α-synuclein seed amplification assays (SAA)—towards biomarker-driven phenotyping in synucleinopathies
Source: NPJ Parkinsons Dis. 2023 May 29;9:79. doi: 10.1038/s41531-023-00519-8 (PMC10226020; doi:10.1038/s41531-023-00519-8)
Supplement: Supplementary file 3 — Reporting Summary [file 41531_2023_519_MOESM3_ESM.pdf]

nature portfolio

Corresponding author(s): Anastasia Kuzkina and Kathrin Doppler

Last updated by author(s): Oct 10, 2022

Reporting Summary

Nature Portfolio wishes to improve the reproducibility of the work that we publish. This form provides structure for consistency and transparency in reporting. For further information on Nature Portfolio policies, see our Editorial Policies and the Editorial Policy Checklist.

Statistics

For all statistical analyses, confirm that the following items are present in the figure legend, table legend, main text, or Methods section.

n/a	Confirmed	
☐	☒	The exact sample size (*n*) for each experimental group/condition, given as a discrete number and unit of measurement
☐	☒	A statement on whether measurements were taken from distinct samples or whether the same sample was measured repeatedly
☐	☒	The statistical test(s) used AND whether they are one- or two-sided<br>*Only common tests should be described solely by name; describe more complex techniques in the Methods section.*
☒	☐	A description of all covariates tested
☒	☐	A description of any assumptions or corrections, such as tests of normality and adjustment for multiple comparisons
☐	☒	A full description of the statistical parameters including central tendency (e.g. means) or other basic estimates (e.g. regression coefficient) AND variation (e.g. standard deviation) or associated estimates of uncertainty (e.g. confidence intervals)
☐	☒	For null hypothesis testing, the test statistic (e.g. *F*, *t*, *r*) with confidence intervals, effect sizes, degrees of freedom and *P* value noted<br>*Give P values as exact values whenever suitable.*
☒	☐	For Bayesian analysis, information on the choice of priors and Markov chain Monte Carlo settings
☒	☐	For hierarchical and complex designs, identification of the appropriate level for tests and full reporting of outcomes
☒	☐	Estimates of effect sizes (e.g. Cohen's *d*, Pearson's *r*), indicating how they were calculated

Our web collection on statistics for biologists contains articles on many of the points above.

Software and code

Policy information about availability of computer code

Data collection	No specific code was used to collect data.
Data analysis	RStudio 2022.02.0 Build 443, Package "Comparegroups" Version 4.5.1

For manuscripts utilizing custom algorithms or software that are central to the research but not yet described in published literature, software must be made available to editors and reviewers. We strongly encourage code deposition in a community repository (e.g. GitHub). See the Nature Portfolio guidelines for submitting code & software for further information.

Data

Policy information about availability of data

All manuscripts must include a data availability statement. This statement should provide the following information, where applicable:

- Accession codes, unique identifiers, or web links for publicly available datasets
- A description of any restrictions on data availability
- For clinical datasets or third party data, please ensure that the statement adheres to our policy

The raw data of RT-QuIC assays is included in the Supplementary Table 1. The full detailed demographic and clinical dataset is not included to protect patient privacy but could be made available from the corresponding authors upon reasonable request.

Human research participants

Policy information about studies involving human research participants and Sex and Gender in Research.

Reporting on sex and gender	Only sex is being reported as documented in the hospital records. We did not collect data on gender as we were assessing biological markers of disease.
Population characteristics	The PD cohort was not significantly older than controls. Sex distribution was in favor of male sex, in accordance with the population prevalence of PD and RBD.
Recruitment	Participants were recruited from the in- and outpatient departments of the University Hospital of Wuerzburg (PD and control subjects) and Cologne (RBD).
Ethics oversight	Ethical review board of the University of Wuerzburg and of the University of Cologne.

Note that full information on the approval of the study protocol must also be provided in the manuscript.

Field-specific reporting

Please select the one below that is the best fit for your research. If you are not sure, read the appropriate sections before making your selection.

☒ Life sciences ☐ Behavioural & social sciences ☐ Ecological, evolutionary & environmental sciences

For a reference copy of the document with all sections, see nature.com/documents/nr-reporting-summary-flat.pdf

Life sciences study design

All studies must disclose on these points even when the disclosure is negative.

Sample size	This is a pilot exploratory study. Sample size calculation is based on the main study subgroups: PD vs control. In the previous RT-QuIC studies of skin biopsies we saw effect sizes (d) between 0.82-2.66 (Kuzkina et al 2021). Sample size calculation based on the available data resulted in the estimate of 25 participants in PD vs controls groups. See Methods section for more details on sample size calculation.
Data exclusions	Pre-established exclusion criteria - patients not reaching a clinical diagnostic certainty of established PD (MDS criteria). 3 PD participants had to be excluded upon thorough review of clinical data as their diagnosis did not fulfill the required diagnostic certainty.
Replication	We saw an excellent the reproducibility of replicates (4), therefore we could apply very stringent criteria for sample positivity - all 4 replicates had to be positive for the sample to be considered positive. Re-tested samples (see detailed reasons for retesting in Methods) showed consistent results.
Randomization	not relevant
Blinding	Patient samples were coded. ENT (nasal brushing acquisition) and laboratory technician (RT-QuIC reaction) were blinded to the patient diagnosis. Samples were deemed positive or negative based on the set criteria (all 4 replicates crossing the 10% fluorescence threshold) in an automated way.

Reporting for specific materials, systems and methods

We require information from authors about some types of materials, experimental systems and methods used in many studies. Here, indicate whether each material, system or method listed is relevant to your study. If you are not sure if a list item applies to your research, read the appropriate section before selecting a response.

Materials & experimental systems

n/a	Involved in the study	
☒	☐	Antibodies
☒	☐	Eukaryotic cell lines
☒	☐	Palaeontology and archaeology
☒	☐	Animals and other organisms
☐	☒	Clinical data
☒	☐	Dual use research of concern

Methods

n/a	Involved in the study	
☒	☐	ChIP-seq
☒	☐	Flow cytometry
☒	☐	MRI-based neuroimaging

Clinical data

Policy information about clinical studies

All manuscripts should comply with the ICMJE guidelines for publication of clinical research and a completed CONSORT checklist must be included with all submissions.

Clinical trial registration	this study is not an RCT (randomized clinical trial)
Study protocol	Protocol 5/19-am reviewed by Ethical review board of the University of Würzburg
Data collection	Recruited at in- and outpatient neurology departments in University Hospitals Würzburg and Cologne between 2018 and 2022.
Outcomes	N/A
